# Alpha-band fluctuations represent behaviorally relevant excitability changes as a consequence of top–down guided spatial attention in a probabilistic spatial cueing design

**DOI:** 10.1162/imag_a_00312

**Published:** 2024-10-15

**Authors:** Christopher Gundlach, Norman Forschack, Matthias M. Müller

**Affiliations:** Experimental Psychology and Methods, Faculty of Life Sciences, Wilhelm-Wundt Institute for Psychology, Universität Leipzig, Leipzig, Germany

**Keywords:** spatial attention, alpha-band activity, steady state visual evoked potentials, vision, reaction times

## Abstract

Spatial attention is a key function enabling the selection of relevantinformation and meaningful behavioral responses and is likely implemented bydifferent neural mechanisms. In previous work, attention led to robust butuncorrelated modulations of Steady-State-Visual-Evoked-Potentials (SSVEPs) as amarker of early sensory gain and visual as well as motor alpha-band activity. Weprobed the behavioral relevance of attention-modulated trial-by-trialfluctuations of these measures. For this purpose, in an experiment with aclassical probabilistic visuospatial attention cueing task, ato-be-discriminated target stimulus was validly, neutrally, or invalidly cued,while behavioral responses and EEG were recorded. Single-trial flicker-drivenSSVEPs, visual and motor alpha-band activity were measured and the relationshipbetween their amplitudes and reaction times was modeled via Bayesian regressionmodels, respectively. We replicated previous findings that these neural measuresand behavioral responses were overall modulated by the attentional cue. Beyondthat, SSVEP amplitudes were not associated with behavior, while single-trialalpha-band amplitudes were predictive of reaction times: For trials with a validor neutral cue, lower visual and motor alpha-band amplitudes measuredcontralateral to the target in the cue–target interval were associatedwith faster responses (and for valid cues also higher amplitudes ipsilateral tothe target). For invalid cues, which required attentional reallocating to theuncued side, no such relationship was found. We argue that behavioral relevanceof alpha-band modulations is a consequence but not a mechanism oftop–down guided spatial attention, representing neural excitability incortical areas activated by the attentional shift.

## 1 Introduction

Spatial attentional selection is a key function in neural processing aiding theselection and prioritization of information relevant for a meaningful interactionwith the environment. One of the fundamental outcomes of the deployment of spatialattention is behavioral benefits for stimuli presented at attended locations andbehavioral costs for stimuli presented at unattended locations (Carrasco, 2011;Posner,1980). This is seen in faster and more accurate responses, enhanceddetection to weaker and less salient stimuli and higher contrast sensitivity orspatial acuity (Carrasco, 2011). Differentneural measures were found to be sensitive to visual spatial attention modulation:Early components of EEG-recorded responses to briefly presented either attended orunattended visual stimuli such as the P1 or N1 were amplitude modulated by attention(Hillyard & Anllo-Vento, 1998;Hillyard et al., 1998;Luck, 1995;Luck et al.,1997,^2000^;Marzecová et al., 2018;Slagter et al., 2016). The earlier findings led to thesensory gain control hypothesis of attention, posing that neural activity associatedwith the processing of stimuli is modulated in amplitude by attention and that thisgain control may be implemented at early stages of visual processing (seeHillyard et al., 1998). The sustained nature ofthis attentional selection and its related enhanced stimulus processing was madeexplicit by employing frequency tagged stimuli that evokedSteady-State-Visual-Evoked-Potentials (SSVEPs). SSVEPs are evoked oscillatorysignals that follow the frequency of the tagged stimulus, respectively (Adrian & Matthews, 1934;Norcia et al., 2015;Regan, 1989), and their generators were found in early visual areas ofthe human brain (Boylan et al., in press;Hillyardet al., 1997;Moratti et al.,2023;Müller, Picton, et al.,1998;Müller et al., 1997;Pastor et al., 2007). Crucially, it wasfound that when such flickering stimuli were spatially attended, flicker-evokedSSVEP amplitudes were increased for the attended as compared with the unattendedstimulus, in line with the sensory gain control hypothesis (Di Russo et al., 2001;Kim et al., 2007;Müller &Hillyard, 2000;Müller, Picton, etal., 1998;Müller,Teder-Sälejärvi, et al., 1998).

Activity in the alpha band, measurable across various sensory regions (Haegens et al., 2015) and animal species (Buzsáki et al., 2013), is another neuralmeasure that was found to be robustly modulated by attentional demands. Invisuospatial attention, it was repeatedly found that alpha-band power was lowercontralateral to the attended as compared with the unattended side (Antonov et al., 2020;Bauer et al., 2014;Bollimunta et al.,2011;Capotosto et al., 2009;Foster et al., 2017;Gundlach et al., 2020;Händel et al., 2011;Keefe& Störmer, 2021;Kelly etal., 2006;Liu et al., 2022;Lobier et al., 2018;Samaha et al., 2016;Sauseng et al., 2005;Siegel et al.,2008;Slagter et al., 2016;Sokoliuk et al., 2019;Thut et al., 2006;Voytek et al., 2017;Worden et al.,2000;Zhigalov & Jensen,2020). Thus, compared with SSVEPs, alpha-band modulations show theopposite pattern (seeKeitel et al., 2019).Besides visual parieto-occipital alpha-band activity, alpha-band activity recordedover somato-motor regions (often labeled mu-alpha activity) also showed task-relatedmodulations. Specifically, a decrease of motor/mu-alpha amplitudes seemed to beassociated with task-related motor preparatory activity (Babiloni et al., 2008;Brinkman et al., 2014,^2016^;Deiber et al., 2012;Maeder et al., 2012;McFarland et al., 2000;Pfurtscheller& Neuper, 1997;Stolk et al.,2019). These results led to the idea that alpha-band activity representsa marker or even a mechanism for altering neural processing by suppressing orfacilitating neural excitability in phases of high or low alpha amplitude,respectively (Jensen & Mazaheri, 2010;Klimesch et al., 2007;Mathewson et al., 2011). Thus alpha-band activity mayeven be relevant for instantiating attentional selection by changing the neuralexcitability in neural populations representing the attended stimulus or feature(Foxe & Snyder, 2011;Peylo et al., 2021).

Importantly, in recent work, we and others found that SSVEP and visualparieto-occipital alpha-band amplitude modulations did not correlate (Antonov et al., 2020;Gundlach et al., 2020;Nuttall et al.,2022;Zhigalov & Jensen,2020), pointing toward that these two measures represent differentmechanisms by which attentional selection of stimulus processing is implemented,which is well in line with the idea that attention does not represent a unitarymechanism but may be implemented at different processing levels (Buschman, 2015;Hommel etal., 2019;Luo & Maunsell,2015;Maunsell, 2015). While theseprevious studies investigated the relationship between SSVEP and visual alphaamplitudes, the behavioral consequences of their attentional modulation were notaddressed and remained elusive. Whereas in the auditory domain some efforts weremade to elucidate the relationship between alpha-band activity, sensory signaltracking, and behavior (Tune et al., 2021),in the visual domain, this relationship—to the best of ourknowledge—has not been addressed yet.

In order to understand the potentially functional role of the two neural signals, inthe present study, we examined the relationship between modulations of alpha-bandactivity, SSVEP amplitudes, and behavior in the context of spatial attention.Crucially, it is well known that attentional states fluctuate over time (Adam & deBettencourt, 2019;Esterman & Rothlein, 2019;Guilford, 1927;Rosenberg et al., 2015;Yamashita et al., 2021). Harnessing this idea, we assumed thatvariations in behavior related to fluctuations in attention should be associatedwith fluctuations in these neural measures, if they were relevant for attentionalselection. For this purpose, we used a probabilistic Posner spatial cueing task(Posner, 1980), in which SSVEP evokingflickering disks were presented on each side of the screen. Spatial attention wasmanipulated by a cue in the form of an arrow indicating the most likely side onwhich a target will be presented prompting to shift attention to the left or rightside of the screen in order to be able to rapidly respond to it by pressing a button(see below). The cue either validly, invalidly, or neutrally (pointing toward bothstimuli) indicated the side of the upcoming target within the flickering stream ofthe disks. Participants had to discriminate the position of a transiently presenteddarker arc (upper or lower half and left or right side) at either the left or rightstimulus in each trial by pressing one of four buttons on the keyboard that wereassigned to the respective position within the two disks. We instructed our subjectsto respond with the left hand to left targets and with the right hand to targets onthe right side. With this design, we were able to concurrently measure and analyzedifferent neural signals on a trial-by-trial basis that had been associated withselective spatial attention in a plethora of studies: stimulus evoked SSVEPs as amarker of early sensory gain, visual and motor alpha-band activity, and behavioralperformance. We expected to replicate the typical behavioral pattern with fasterresponses for validly compared with invalidly cued targets (Carrasco, 2011;Posner,1980).

As outlined above, relative to precue baseline, SSVEP amplitudes should increase forthe cued as compared with the uncued stimulus, visual alpha-band amplitudes shouldbe higher ipsilateral to the cued as compared with the uncued side, and motoralpha-band amplitudes should be reduced contralateral to the cued response hand.However, in one of our previous spatial cueing studies (Gundlach et al., 2020), we found considerable variationsand variance in these measures across trials. Indeed, on a substantial number oftrials, the lateralization pattern for visual alpha was either reversed, that is,more alpha at electrodes contralateral to the cued side, or the differences betweenthe cortical hemispheres were negligible. Interestingly, the particular alpha-bandpattern had no influence on SSVEP amplitudes (see also below). As the previous studyfocused on the relationship between these neural measures, by design we did notexplicitly test the influence of these varying neural signals on reaction times. Inthe current study, designed to test this relationship, we hypothesized that fasterresponses to validly cued targets should be related to higher SSVEP amplitudesand/or lower visual alpha contralateral and higher visual alpha amplitudesipsilateral to the cued side, as well as lower motor alpha amplitudes for theresponse hand in the cue–target interval. In the same manner, the questionarises whether these neural fluctuations in the cue–target interval arepredictive of a faster shift of the attentional spotlight to the other side, andthus, of faster reaction times for invalid trials, during which subjects do not“know” that a target will occur at the uncued side. In contrast to thepattern we hypothesized for trials with a valid cue, for trials with an invalid cue,one would expect the opposite pattern of alpha-band activity in thecue–target interval: lower alpha at ipsilateral and higher alpha amplitudesat contralateral electrodes relative to the cue for faster responses. The sameshould be true for SSVEP amplitudes (i.e., higher for the uncued stimulus atipsilateral sites), if they contributed to the speed of responses. For motor alphawe would expect higher motor activation (i.e., lower amplitudes) for the motorcortex, ipsilateral to the cued side. To test the contributions of these EEGmeasures to the variance of reaction time, we employed Bayesian multilevel modelsbased on single-trial data (seeSection 2).

This Bayesian modeling approach helped to uncover the contribution of SSVEP, visualand motor alpha-band activity to behavioral responses. As attentional modulationshave been shown to affect these measures independently (Antonov et al., 2020;Gundlach et al., 2020;Nuttall et al.,2022;Zhigalov & Jensen,2020), one may assume that they also contribute to reaction timevariability in different ways. Thus, SSVEP, visual, and motor alpha-band activitymay reflect different stages in a hierarchical processing architecture. Based onprevious suggestions, it might well be possible that alpha-band activity reflects agating mechanism at later processing stages (Peyloet al., 2021;Zhigalov & Jensen,2020) that operates independently from stimulus processing at earlyvisual stages. Thus, alpha may be more closely related to the sensory readoutrelevant for behavioral performance, while SSVEPs might reflect early visualrepresentation in a narrow sense not directly affecting the speed of the behavioraloutput.

## 2 Materials and Methods

### 2.1 Participants

In total, 28 participants (18 females, mean age 23.82 years; range 18 to 34) tookpart in the experiment. After being informed about the nature and the generalaim of the experiment, all participants gave written informed consent.Participants either received class credits or were financially reimbursed (10€ per hour). The study was designed and conducted according to theDeclaration of Helsinki and the local ethics committee (298/17-ek,Ethik-Kommission an der Medizinischen Fakultät der UniversitätLeipzig).

### 2.2 Stimuli, procedure, and task

Visual stimulation was created with custom scripts using the Psychophysicstoolbox 3 (Brainard, 1997;Kleiner et al., 2007), Matlab R2018 (TheMathWorks, Natick, MA) running in an Ubuntu environment. Stimuli were presentedvia a PROPixx DLP LED projector (VPixx Technologies Inc., Canada) displayingimages with a resolution of 960-by-540 pixels at a refresh rate of 480 Hz,projecting an image on a screen situated 120 cm in front of the subject.

The stimulus display was comparable with a display used in a previous study(Gundlach et al., 2020) and consistedof two rings with an outer and inner diameter of 5.4° and 3.2° ofvisual angle, presented 7.4° left and right, and 4.7° below acentrally presented gray fixation cross (luminance 420 cd/m²) with aheight and width of 0.5° of visual angle on a dark gray background(luminance ~23 cd/m², seeFig. 1).Both rings flickered between background gray and bright gray (luminance 420cd/m²) at 24 Hz for the left stimulus and at 21.818 Hz for the rightstimulus.

After the introduction to the study aim and experimental procedure, the EEG wasset up and participants were subsequently seated comfortably in anelectromagnetically shielded and acoustically damped chamber. Participants thenran a few training trials to practice the precue and the main task (see below),got familiarized with the stimulus material, and practiced to blink only duringthe intertrial interval and to fixate the fixation cross throughout the entiretrial.

In a given trial, during the precue period, participants attended to the fixationcross to discriminate transient increases (press “L” with theright index finger) or decreases (press “S” with the left indexfinger) of either the vertical or horizontal arm by 6.7 to 33.3% of size for aduration of 100 ms. In 20% of the trials, a single precue event was randomlypresented in a time window between 500 ms after flicker onset and 400 ms beforecue presentation. After a jittered precue interval of 1,500 to 2,000 ms, toavoid temporal expectation effects for the cue, the fixation cross changed colorof the left and/or right arm to cue the to-be-attended side and remained presentfor the postcue period of 3,250 ms. Following this cue, a target stimulus on oneof the rings was presented in each trial. The majority (90%) of targets werepresented in a time window from 1,500 to 2,500 ms after cue onset (regulartrials that entered the analysis), and 10% of the targets were presented inbetween 300 and 1,499 ms after the cue. As targets were presented throughout thetrial and even close to the cue, we expected participants to shift theirattention right after the cue and maintain attention at the cued side in asustained manner anticipating the target stimulus. The presentation of themajority of target stimuli 1,500 ms after the cue (regular trials) allowed us toanalyze a time window from 500 to 1,500 ms after the cue, for which spatialattentional shifts should have been accomplished and sustained attentionestablished (Gundlach et al., 2020;Müller, Picton, et al., 1998;Müller, Teder-Sälejärvi, etal., 1998). Importantly, given that this time window was without anytarget stimulus in trials that entered the analysis, we were able to analyzeSSVEP and alpha-band activity that was not distorted by an evoked potentialelicited by a target event.

The target stimulus was a transient 150 ms long luminance decrease (to 270cd/m²) of an arc with a width of 8° in either the left or rightring and participants had to discriminate and respond to whether this arc waspresented in the upper or lower half of the left stimulus by pressing“Q” or “S” or in the upper and lower half of theright stimulus by pressing “P” or “L” on a standardQWERTZ keyboard. Importantly, subjects responded with the hand of the targetposition. In other words, when a target occurred on the right side, theyresponded with their right hand and vice versa to allow for the analysis ofmotor-related alpha-band amplitudes (see below). Target positions were evenlydistributed between the left and right rings and upper and lower halves.Responses were registered in a time window between 200 and 1,200 ms followingtarget onset. As in a typical Posner paradigm (Posner, 1980), the cue indicated the appearance of the targetstimulus in a nondefinite but probabilistic way. From a total of 700 trials, 140trials were neutral, that is, the cue pointed toward both stimuli. Of theremaining 560 trials in which the cue pointed either to the left or rightstimulus, 420 valid trials (75%) correctly indicated the side of the target,while in 140 invalid trials (25%), the target was presented at the side oppositeto the cue. Single trials were separated by an intertrial interval of 1,000 msand were presented in 14 blocks with self-paced breaks in between.

### 2.3 Electrophysiological data recording and preprocessing

EEG was measured from 64 Ag/AgCl electrodes mounted in an elastic cap with anActiveTwo Amplifier (BioSemi, Amsterdam) at a sampling rate of 512 Hz with alow-pass filter of 104 Hz and stored for later offline analysis. Two electrodeswere placed vertically above and below the right eye, and two were placedhorizontally at the canthi of both eyes to allow measuring vertical andhorizontal eye movements and blinks.

For offline data processing and analysis, the EEGLAB toolbox (Delorme & Makeig, 2004) and custom-developedMatlab scripts (The MathWorks, Natick, MA) were used. As a first step,continuous data were resampled to 256 Hz and epoched from -2,000 to 4,250 msrelative to cue onset. This relatively large time window was selected to allowfor an analysis of electrophysiological single-trial data locked to the cue andtarget presentation, respectively (see below)^*^. In the following, weidentified artifacts and contaminated data (see below) in the time window 1,000ms before and 3,250 ms after the cue to allow for an additional pretarget windowanalysis (see below).

First, linear trends were removed and blinks identified via an adaptive thresholdprocedure run for bipolarized vertical eye channels. Horizontal eye movementswere identified as amplitudes exceeding 25 µV (representing eye movementsof around 2° visual angle) in the bipolarized horizontal eye channels(blinks: average number of trials discarded per subject:*M*= 35.536;*SD*= 35.860; eye movements: averagenumber of trials discarded per subject:*M*= 164.429;*SD*= 96.653). By implementing the“statistical control of artifacts in dense array EEG/MEG studies”(Junghöfer et al., 2000),single artifact-contaminated channels were identified based on statisticalparameters and were spline interpolated. In case of more than 15 noisy electrodechannels per trial, the entire trial was rejected. On average, 31.000 trials(*SD*= 26.369) were discarded and 3.722 channels pertrial (*SD*= 0.899) interpolated. Overall, for eachsubject on average 260.786 valid (*SD*= 64.741), 90.857invalid (*SD*= 19.357), and 88.000 neutral trials(*SD*= 23.051) entered the analysis. Preprocessed,artifact-free data were transformed to reference-free scalp current densities(SCDs) to represent data with more distinct local signal maxima (Ferree, 2006;Kayser,2009;Kayser & Tenke,2006;Müller et al.,2018).

### 2.4 Data analysis

The general aim of the current study was to examine the relationship betweenparieto-occipital visual alpha-band, motor alpha-band activity, SSVEPs, andbehavior in a visuospatial attention paradigm after the cue and before thepresentation of a target. As mentioned above, only those trials were consideredfor analysis, in which a target was presented at least 1,500 ms after the cue(called regular trials above), catch trials were all excluded.

#### 2.4.1 Behavioral analysis

Button presses within a time window between 200 and 1,200 ms after targetonset were considered as responses in the postcue period. The same windowapplied to events at the fixation cross, before the cue. Correct, incorrect,or missed responses to increases or decreases were analyzed descriptively.For targets after the cue, responses were correct if participants correctlydiscriminated the position (upper vs. lower half and left vs. right side).The average percentage of correct responses for each cueing condition wastested with a conventional repeated-measures ANOVA comprising the factorsVALIDITY and SIDE, using the afex package (Singmann et al., 2020) in*R*(R Core Team, 2016). For the ANOVA, degrees offreedom were Greenhouse–Geisser (GG) corrected in case of violationof the sphericity assumption. For this ANOVA model, post-hoc pairwisecomparisons between the modeled marginal mean reaction times of thedifferent levels of the factor VALIDITY were tested via the emmeans package(Lenth, 2023).*p*-Values were corrected for multiple comparisons via Holmcorrection (Holm, 1979) andCohen’s*d*-values and respective 95% confidenceintervals (95%-CI) will be reported, calculated with the population standarddeviation defined as the root mean square value of the residual standarddeviations of all factor levels via function*eff_size*ofthe emmeans package. We expected to replicate the common finding of fasterresponses for target events in validly compared with invalidly cued sideswith neutral cues in between (Carrasco,2011;Posner, 1980).

For examination of the relationship between electrophysiological signals andbehavior, we were particularly interested in reaction times. As a firststep, mirroring the analysis of the percentage of correct response,condition-averaged reaction times were tested with a conventionalrepeated-measures ANOVA comprising the factors of CUE_VALIDITY and SIDE andHolm-corrected post-hoc pairwise comparisons of the marginal means.

#### 2.4.2 Analysis of electrophysiological data

##### 2.4.2.1 Analysis of SSVEPs

As a first omnibus test, we tested whether we can replicate thewell-known finding of an SSVEP amplitude increase, relative to theprecue period, for the to-be-attended compared with the to-be-ignoredside/ring (Gundlach et al., 2020;Müller & Hillyard,2000). But of particular interest was whether or notsingle-trial SSVEP amplitudes and reaction times are related to eachother (with higher amplitudes found in trials with lower reactiontimes). In order to extract single-trial SSVEPs, we used a recentlydeveloped spatial filtering approach by which the SSVEP signal isextracted from an optimally weighted sum of all EEG sensors based onRhythmic Entrainment Source Separation (RESS) (Cohen & Gulbinaite, 2017). Thisprocedure derives spatial RESS filters with an optimized representationof the (SSVEP-) signal over noise, individually determined for eachsubject and frequency of interest. For the implementation of thisprocedure, one spatial filter for each signal was derived for which thesignal component was centered at the respective SSVEP frequency (24 and21.818 Hz) with a bandwidth of 0.5 Hz, while noise was defined asspectral components ±1.5 Hz apart from the signal with abandwidth of 1.5 Hz. For a more numerically stable estimation of thespatial filter estimation, the covariance matrix was regularized byadding 1% of the mean of its eigenvalues to its diagonal (Gulbinaite et al., 2019;Schettino et al., 2020).Individually filter-projected data (seeFigure 3for a topographical representation of the filterweights) were then used for extracting SSVEP amplitudes.

In a first analysis, we were interested in the general pattern ofattentional modulation for trials of the different cueing conditions(valid, neutral, invalid). For this purpose, RESS-filter-projectedsingle-trial data for each SSVEP and experimental condition were FFTtransformed (zero-padding to 16,384 datapoints) from a precue (-1,000 to0 ms before the cue) and postcue time window (500 to 1,500 ms after thecue). SSVEP amplitudes for each stimulus, condition, trial, and timewindow were then derived by averaging the amplitude values in a range of±0.1 Hz around the respective SSVEP frequency and averagingacross trials of each condition. As exact SSVEP peak frequencies mayslightly differ between participants and SSVEP, amplitude changes withinthe FFT time window may lead to amplitude changes of the SSVEP frequencysidebands (Cohen & Gulbinaite,2017), by zero-padding the data and summing amplitudes acrossa frequency range, we made sure to capture SSVEP amplitudes for eachparticipant. As previously reported (Gundlach et al., 2020), precue SSVEP signals are seen todepict the representation of the unattended flickering rings in earlyvisual areas (i.e., while attention was allocated to the fixationcross). This baseline measure allows to measure cortical facilitationthrough attention after the cue. To this end, percentual changes ofSSVEP amplitudes from the pre- to postcue time window were calculated.These values were then collapsed across stimuli and conditions in a waythat they represent pre- to postcue SSVEP amplitude modulations for thecued, uncued, or neutrally cued stimulus. Potential differences betweenthese SSVEP modulations were then tested via a one-way repeated measuresANOVA and Holm-corrected post-hoc pairwise comparisons between modeledmarginal mean SSVEP modulations for the cued, uncued, and neutrally cuedrings, as described above. In addition, for each factor level, marginalmean SSVEP modulations were tested against zero to estimate pre- topostcue modulations per se (Holm corrected for multiple comparisons).Cohen’s*d*-values were calculated with thepopulation standard deviation defined as the root mean square value ofthe residual standard deviations of all factor levels.

##### 2.4.2.2 Analysis of alpha-band activity

For the analysis of visual and motor-related alpha-band activity for thedifferent attentional cueing conditions, the analysis rationale wassimilar to the one described above. The basis of this analysis formedsingle-trial data, for which all but the SSVEP-RESS components were backprojected (Cohen & Gulbinaite,2017). To minimize the potential contamination of alpha-bandactivity by subharmonics and intermodulation frequencies of the SSVEPsignals (Labecki et al., 2016;Zemon & Ratliff,1982,^1984^) and allow foran independent analysis of SSVEP and alpha-band activity, the RESScomponent was excluded before back-projection. Single-electrode andsingle-trial data were FFT transformed (zero-padding to 16,384datapoints) for the same analysis windows as for SSVEPs. Visualalpha-band amplitudes (averaged across 8 to 12 Hz) were extracted (andaveraged) for each experimental condition and time window from anelectrode cluster contralateral to the left (P6, P8, P10, PO4, PO8, O2,I2) and right stimuli (P5, P7, P9, PO3, PO7, O1, I1) (electrode clustersidentical toGundlach et al.,2020). As above, percentual pre- to postcue modulations werecalculated and then averaged to depict visual alpha-band modulationscontralateral to the cued, uncued, or neutrally cued stimulus. Of note,in previous literature cue-related modulations were often found in bothhemispheres and it is still under debate whether signals are related tomodulations contra- and ipsilateral to the cued side or signalscontralateral to the cued and the uncued side and thus potentiallyrelated to enhanced target vs. attenuated processing of the nontargetstimulus (Capilla et al., 2014;Orf et al., 2023). In otherwords, while we are referring to alpha-band activity contralateral tothe cued or uncued stimulus, the signal is per se ambiguous, asalpha-band activity contralateral to the cued stimulus can also belabeled as activity ipsilateral to the uncued stimulus for instance.Motor preparatory processes were analyzed in the same manner byextracting motor alpha-band activity (averaged across 9 to 14 Hz) fromtwo electrodes contralateral to the left (C3, CP3) and right (C4, CP4)response hands, respectively. Pre- to postcue motor alpha-bandmodulations contralateral to the cued, uncued, or neutrally cued handwere calculated the same way as above. As for the SSVEP amplitudemodulations, attention-related differences in visual and motoralpha-band amplitude modulations were tested via one-way repeatedmeasures ANOVAs with additional Holm-corrected post hoc pairwisecomparisons based on the estimated marginal means of the ANOVA models.As an important critical test, we analyzed and tested the phasecoherence between electrode sites at lateralized parieto-occipitallocations (see above) and C3/C4 and CP3/CP4 to make sure that alpha-bandsignals measured at these sites have distinct neural generators and arenot mainly based on either volume conduction of the same signal orrepresent a single dipole projecting to central and visual leads (seeSupplementaryMaterial). Results revealed a significant difference in phasebetween these sites (i.e., nonzero phase lag), suggesting thatalpha-band activity recorded from motor as well as parieto-occipitalchannels had different neural generators.

#### 2.4.3 Analysis of the relationship between reaction times, attention, and electrophysiological measures

In the next step, we tested to what extent trial-by-trial fluctuations ofSSVEP and alpha-band amplitudes under sustained attention were predictive toreaction times. Electrophysiological measures and reaction times wereextracted on the single-trial level, as described above. We used absoluteamplitude values of the postcue period from 500 to 1,500 ms after the cueinstead of modulation values used in the ANOVA models above to allow for ananalysis of the postcue signals unbiased by precue amplitude signals andnoise.

Bayesian multilevel models were fit to predict participants’single-trial response times by different combinations and interactions ofpotentially predicting factors. These factors were validity of theattentional cue (Val.), SSVEP amplitude contralateral to side of target(SSVEP), visual alpha-band amplitudes contralateral to side of target(V.alpha_contra_) and ipsilateral to target side(V.alpha_ipsi_), as well as motor alpha-band activitycontralateral to the side of target which was identical to the response hand(M.alpha_contra_). These Bayesian multilevel models were builtand fit using the package*brms*(Bürkner, 2017) running in*R*(R Core Team, 2016). Right skewed reaction timedistributions were modeled as being represented by a shifted log-normaldistribution (Cousineau et al., 2004;Wagenmakers & Brown,2007), seeEquation1.

$$f(x)=\frac{1}{(x-\theta )\sigma \sqrt{2\pi }}e^{-\frac{1}{2}{\left(\frac{\ln(x\;-\;\theta )\;-\;\mu }{\sigma }\right)}^{2}}$$ (1)

with parametersμor “difficulty” specifying the mean of the log-normaldistribution,σor “scale” representing the standard deviation of thelog-normal distribution andθor “shift” specifying the earliest possible response. Thepredictors and their interactions were modeled to affect the parameterμwhileσandθwere set to be fixed and fit to the data. Separately for our differentneurophysiological predictors, the most complex model was fit assuming aninteractive relationship between the factor cue validity and the respectiveneurophysiological measure (i.e., SSVEP, visual alpha-band and motoralpha-band amplitude). The effects of the factor cue validity were allowedto vary across the grouping factor “subjects” (seeTable 1for the notation of thedifferent models). To increase the model convergence by running brms,continuous predictors are internally centered. Coefficient estimates arethen, however, reported on the original scale, facilitating theinterpretation of the coefficients and effects.

**Table 1.** Notation of the different neurophysiological models.

Model	Notation
SSVEP model	RT ~ Val. * SSVEP + (Val. | sub)
Vis. alpha model	RT ~ Val. * V.alpha _contra_ + Val. * V.alpha _ipsi_ + (Val. | sub)
Motor alpha model	RT ~ Val. * M.alpha _contra_ + (Val. | sub)

RT = reaction times, Val. = cue validity, V.alpha= visual alpha contralateral or ipsilateral to ring withtarget, M.alpha = motor alpha contralateral to responsehand, sub = subject.

Model parameters were fit in four chains, each with 2,000 iterationsincluding 1,000 warmup draws, adding to a total of 4,000 postwarmup draws.Model summaries were then checked for model convergence of model parameters(all parameters converged for all models presented here: Rhat values inrange of 1 to 1.02). Separately for the different model families, themarginal effects of the different predictors (e.g., the slope for themodeled relationship between SSVEP amplitudes and reaction times) were thenextracted and interpreted. This was done, first, by using the model with itsfitted model parameters to create posterior distributions of predictedvalues (i.e., reaction times) that also capture the uncertainty of the modelparameters across the model fits of each brms model. For this purpose,posterior predicted values that were expected for the levels of thepredicting factors in the model were extracted separately for all the modeldraws of the respective fitted model (via function epred_draws of the*tidybayes*package as a vignette of thebrms::posterior_epred function). The*emmeans*package thenallowed to extract the posterior distribution of predicted median RTsseparately for the different levels of the predictors of interest and theircredible intervals, which were then taken to plot and examine the marginaleffect of the factor cue validity on reaction times as captured in themodel. Pairwise contrasts between factor levels were calculated to extractthe marginal effect sizes as well as their 95% highest posterior density(HPD) intervals as a measure of model uncertainty, and to evaluate how muchfaster participants responded after valid as compared with invalid cues forinstance. For the continuous neurophysiological predictors, the modeledslopes (and their 95% HPD intervals) were extracted via the functionemmeans::emtrends, to examine how the different single-trial predictorsaffected single-trial reaction times. The 95% HPD was used to estimate theconsistency of the effect of a certain predictor across the model draws. Ifthe slope of the predicted relationship between one of theneurophysiological measures and reaction times was not consistentlydifferent from zero across the model draws (i.e., the 95% HPD contained thezero), we interpreted the effect of this predictor on reaction times as notconsistent/substantial. This allowed us to estimate whether any of thesingle-trial neurophysiological measures was indeed a relevant factor forpredicting reaction times in our experiment.

Additionally, to estimate how much variance was explained in the model andthe respective predictors, Bayesian$R^{2}$values for each model were calculated to estimate the amount of varianceexplained by the predictions of each model relative to the predictions plusvariance of the errors (Gelman et al.,2019). Predictor-specific effects were evaluated by additionallyfitting two basic models and extract Bayesian$R^{2}$values for these: one intercept model, for which reaction time measures weremodeled to differ between subjects (RT ~ 1 + (sub)) and a modelincluding the cue validity as the only predictor (RT ~ Val. + (Val. |sub)). The latter model would correspond to the classical findings (seePosner, 1980) of longer reactiontimes for invalidly and shorter reaction times for validly cued targetsassociated with typical spatial cueing paradigms.

In addition, the same modeling and evaluation pipeline was implemented forSSVEPs, visual alpha, and motor alpha data derived from a different timewindow: the pretarget time window 1,000 ms before target presentation(-1,000 to 0 ms). As the target stimulus was presented at random time pointsafter the presentation of the cue, this additional analysis allowed toestimate the impact of differences in the amplitude levels of theelectrophysiological signals measured directly before target presentation incontrast to the general sustained postcue modulation described above and mayhave picked up behaviorally relevant modulations on a smaller time scale,that is, right before stimulus processing.

## 3 Results

### 3.1 Behavioral results of the precue period

During the precue task, participants responded correctly to 81.862%(*SD*= 11.264), missed 7.832% (*SD*= 7.280), and responded incorrectly to 10.306% (*SD*= 9.391) of the events at the fixation cross. Therefore, hit ratessuggested that participants were compliant with the precue task during whichattention was focused to central fixation.

### 3.2 Behavioral results of the postcue period

We replicated the typical pattern: On average, participants responded faster andwith the highest rate of correct responses following valid cues and much slowerand with the lowest hit rate following invalid cues (seeTable 2andFig. 2).The repeated measures ANOVA for reaction times revealed a main effect for thefactor of VALIDITY (*F*(1.628,43.943) = 20.160,*p*< .001, η_g_² =0.031). Holm-adjusted post hoc pairwise comparisons of the marginal meansrevealed lower reaction times for validly as compared with invalidly cuedtargets (*t*(54) = 6.254,*p*<.001,*d*= 0.422,*95%-CI_d_*= [0.245 0.600]), significant differences between valid and neutral(*t*(54) = 2.173,*p*= .034,*d*= 0.147,*95%-CI_d_*= [0.006 0.288]), and neutral and invalid reaction times(*t*(27) = 4.081,*p*< .001,*d*= 0.276,*95%-CI_d_*= [0.121 0.431]). Presentation side of the target did not affect reactiontimes as neither the main effect of SIDE (*F*(1,27) =0.027,*p*= .870, η_g_² <0.001) nor the interaction VALIDITY X SIDE was significant(*F*(1.596,43.099) = 3.059,*p*=.068, η_g_² = 0.003). Similarly, the analysis ofcorrect responses revealed a main effect of VALIDITY(*F*(1.374,37.087) = 8.919,*p*=.002, η_g_² = 0.040). Again, we found asignificant difference in the marginal means between validly and invalidly cued(*t*(54) = 3.866,*p*< .001,*d *= -0.451,*95%-CI_d_*= [-0.715 -0.181]) and neutrally and invalidly cued targets(*t*(54) = 3.406,*p*= .003,*d*= -0.397,*95%-CI_d_*= [-0.655 -0.139]), while the difference between validly and neutrallycued targets was not significant (*t*(54) = 0.647,*p*= .647,*d*= -0.054,*95%-CI_d_*= [-0.288 0.181]). Again,there was no main effect of SIDE (*F*(1,27) = 0.087,*p*= .770, η_g_² <0.001) and no significant interaction VALIDITY X SIDE(*F*(1.363,36.796) = 0.18,*p*=.770, η_g_² = 0.001).

**Fig. 1.**
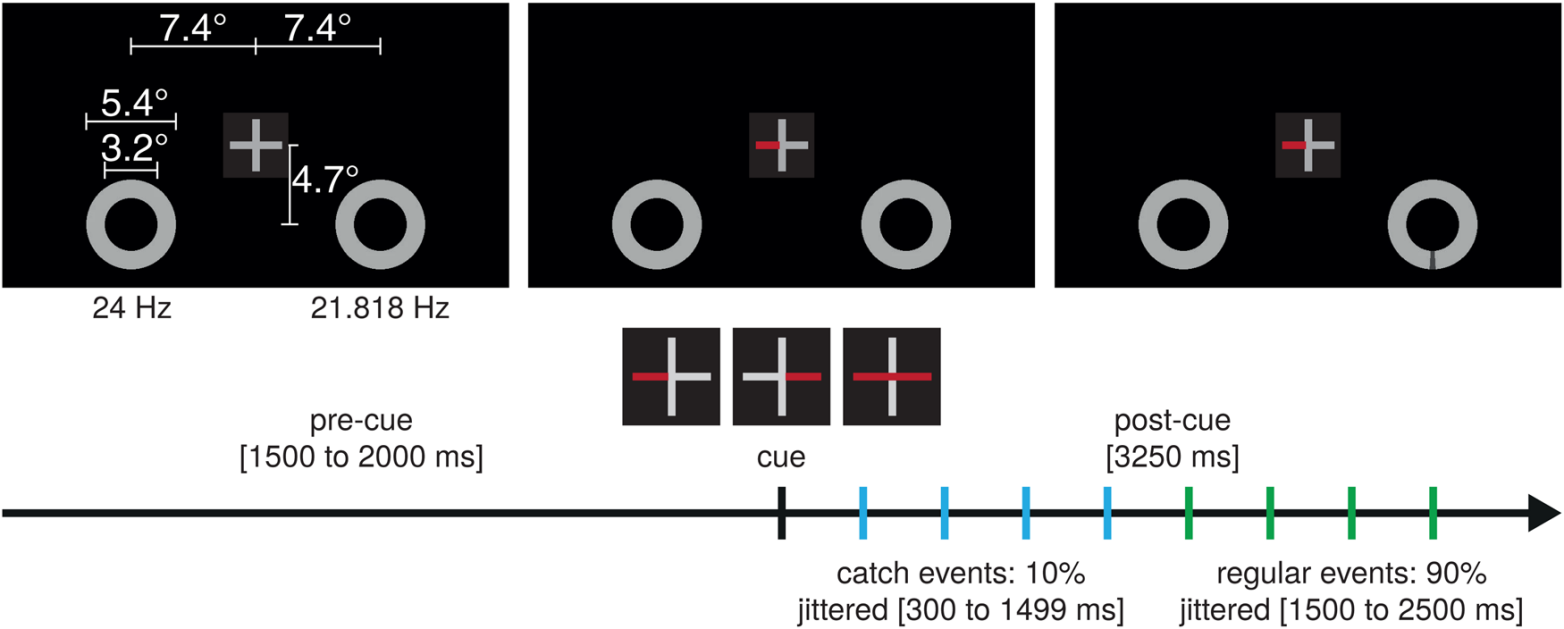
Stimulus display and experimental design of the main experiment.Graphical representation of the stimulus display with a ring presentedleft and right to a centrally located fixation cross. Flickerfrequencies are depicted below the rings. During the precue period, thefixation cross was task relevant as transient infrequent increases ordecreases of the horizontal or vertical arm had to be discriminatedwhile the rings were unattended. Following the baseline precue period(jittered between 1,500 and 2,000 ms), a section of the fixation crosschanged color to indicate on which side the target will be likelypresented and, thus, which side to attend to. Either the left or theright horizontal arm turned red indicating the most probable side of theupcoming target or both arms turned red not providing any information onthe target side probability. Fixation cross enhanced for illustrationpurposes and not to scale. Subsequently, the position (up, down, left,right) of a transient luminance decrease of an arc at one of the rings(target) had to be reported. Target stimuli could be presentedthroughout the trial. Targets of trials that entered the analysis werepresented between 1,500 to 2,500 ms after cue and targets of catchtrials between 300 and 1,499 ms.

**Fig. 2:**
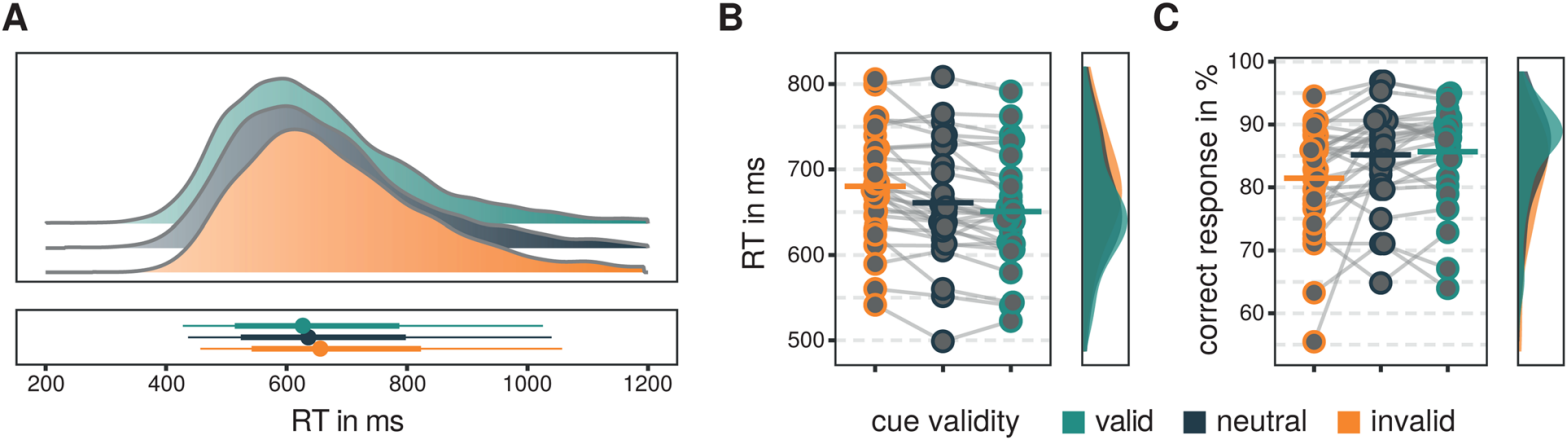
Graphical representation of the behavioral data. (A) Representation ofsingle-trial reaction time density distributions for correct responsesfor all experimental conditions, respectively. Intervals at the bottomrepresent intervals in which 95% (thin line), 66% (bold line), and themedian (circle) of all single-trial reaction times fall. (B)Single-subject trial-averaged reaction times for all cue validitylevels. Dots represent single subjects and horizontal bars represent thegrand mean. Resulting smoothed and normalized distributions arerepresented on the right. (C) Same as (B) but correct response rate in%.

**Table 2.** Behavioral data for the different cueing conditions, respectively.

Cue validity	RT in ms		Correct response rate in %	
	*M*	*SD*	*M*	*SD*
Invalid	680.199	70.373	81.445	10.862
Neutral	660.857	71.685	85.156	8.485
Valid	650.559	66.687	85.658	8.216

### 3.3 General cue-related modulations of SSVEP amplitudes

RESS components exhibited a lateralized topographical distribution for each ofthe two SSVEPs, and Grand Mean FFT amplitude spectra showed distinct amplitudepeaks for the respective frequencies (seeFig.3A).

**Fig. 3.**
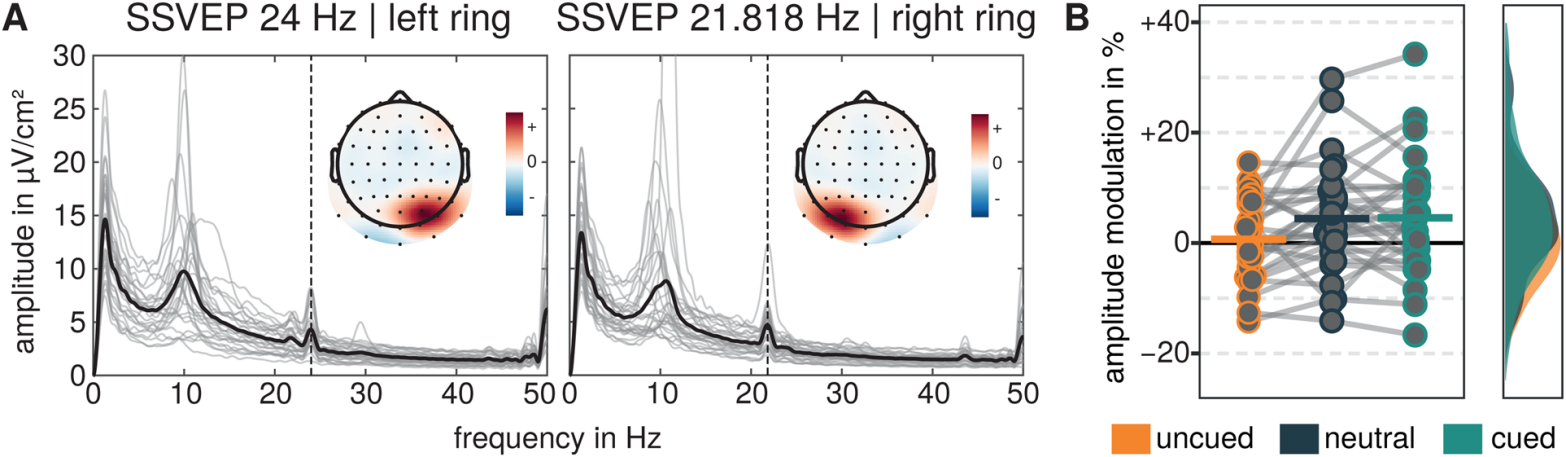
SSVEP Signals and Amplitude Modulations. (A) FFT-derived precue amplitudespectra from single-trial RESS filtered data averaged across allexperimental conditions and trials, separately for RESS filtersdepicting SSVEP signals for left and right stimuli flickering at 24 and21.818 Hz (vertical dashed line). Light gray lines represent theamplitude spectra of single participants and the thick black linerepresents the average. Topographical representations of the weights ofthe individual spatial RESS filters averaged across participants inarbitrary units are represented in each spectra plot. Highest filterweights are found in occipital sensors contralateral to the stimulusside. (B) Pre- to postcue amplitude modulations for SSVEPs evoked bycued, uncued, and neutrally cued flickering rings. Dots represent singlesubjects and horizontal bars represent the grand mean. Resultingsmoothed and normalized distributions are represented on the right.

The analysis of SSVEP amplitude modulations (seeFig. 3B) revealed differences between cueing conditions (seeFig. 3) as revealed by a main effect of thefactor cue in a repeated measures ANOVA (*F*(1.944,52.475)= 4.829,*p*= .013, η_g_²= 0.036). Planned Holm-corrected comparisons of the marginal meansrevealed significantly positive SSVEP amplitude modulations for the cued side(*M*= 4.558 %,*SE*= 1.794,*t*(41.436) = 2.541,*p*= .045,*d*= 0.480,*95%-CI_d_*= [0.077 0.884]) and the neutral condition (*M*=4.426 %,*SE*= 1.794,*t*(41.436) =2.468,*p*= .045,*d*= 0.466,*95%-CI_d_*= [0.064 0.869]), while therewas no SSVEP modulation for the uncued side relative to the precue baseline(*M*= 0.699 %,*SE*= 1.794,*t*(41.436) = 0.390,*p*= .699,*d*= 0.074,*95%-CI_d_*= [-0.308 0.456]). While SSVEP amplitude modulations for neutrally cuedand cued sides did not differ significantly (*M*= 0.132%,*SE*= 1.410,*t*(54) = 0.093,*p*= .926,*d*= -0.014,*95%-CI_d_*= [-0.312 0.284]), they bothdiffered from the modulation for the uncued side (uncued vs. cued:*M*= 3.859 %,*SE*= 1.410,*t*(54) = 2.737,*p*= .025,*d*= -0.407,*95%-CI_d_*= [-0.725 -0.089], uncued vs. neutrally cued:*M*=3.728 %,*SE*= 1.410,*t*(54) =2.644,*p*= .025,*d*= 0.393,*95%-CI_d_*= [-0.709 -0.076]).

### 3.4 General cue-related modulations of alpha-band activity

Visual alpha-band activity recorded at two lateralized occipital electrodeclusters revealed cue-related amplitude modulations in a time window of 500 to1,500 ms after the cue relative to -1,000 to 0 ms before the cue (seeFig. 4).

**Fig. 4.**
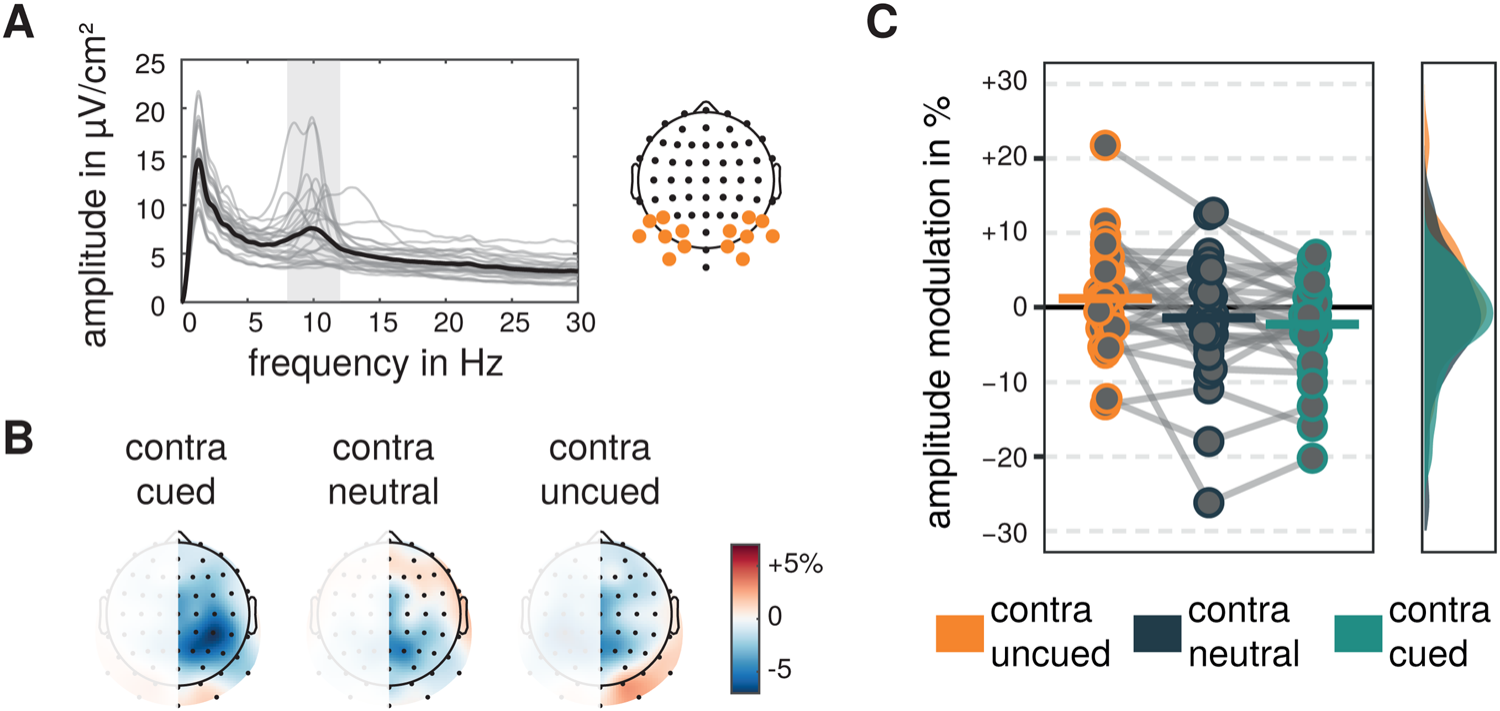
Visual alpha-band signals and amplitude modulations. (A) FFT-derivedprecue amplitude spectra derived from single-trial data recorded at twolateral electrode clusters averaged across all experimental conditionsand trials. Light gray lines represent amplitude spectra of singleparticipants and the thick black line represents the average. Frequencyrange of interest is shaded in gray. (B) Topographical representation ofaverage pre- to postcue visual alpha amplitude modulations for thehemisphere contralateral to the cued, uncued, or neutrally cuedstimulus. Modulations were averaged for left and right stimuli and cuesand collapsed across hemispheres. (C) Pre- to postcue amplitudemodulations for visual alpha-band activity contralateral to cued,uncued, and neutrally cued sides. Dots represent single subjects andhorizontal bars represent the grand mean. Smoothed and normalizeddistributions are represented on the right.

**Fig. 5.**
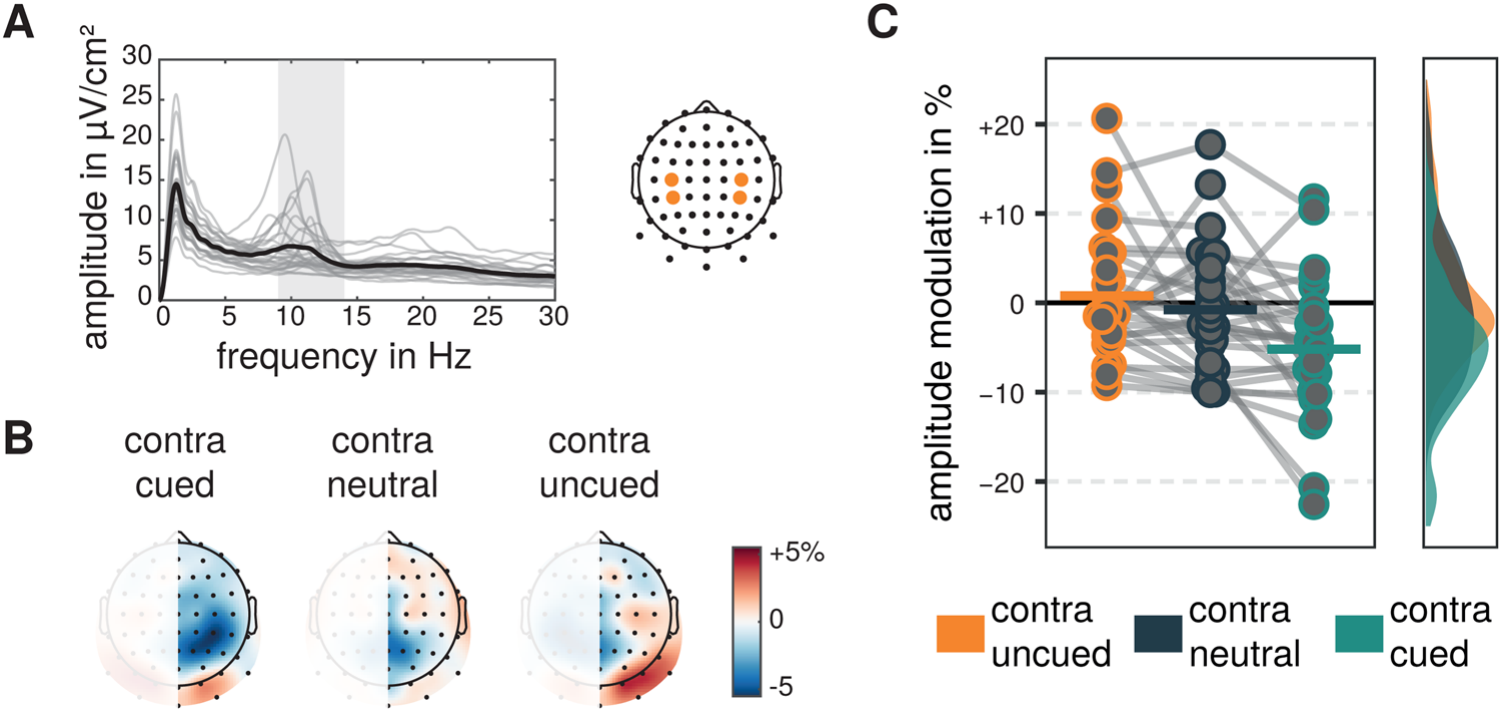
Motor alpha-band signals and amplitude modulations. (A) FFT-derivedprecue amplitude spectra derived from single-trial data recorded at twolateral motor-relevant electrode clusters averaged across allexperimental conditions and trials. Light gray lines represent amplitudespectra of single participants and thick black line represents theaverage. Frequency range of interest is shaded in gray. (B)Topographical representation of average pre- to postcue motor alphaamplitude modulations for the hemisphere contralateral to the cued,uncued, or neutrally cued stimulus (i.e., before motor execution).Modulations were averaged for left and right stimuli and cues andcollapsed across hemispheres. (C) Pre- to postcue amplitude modulationsfor motor alpha-band activity contralateral to cued, uncued, andneutrally cued stimuli. Dots represent single subjects and horizontalbars represent the grand mean. Smoothed and normalized distributions arerepresented on the right.

For visual alpha-band activity, the repeated measures ANOVA revealed a maineffect of the factor cue (*F*(1.421,38.374) = 4.697,*p*= .025, η_g_² =0.039). Holm-corrected comparisons of the marginal means revealed significantlylower visual alpha amplitude modulations contralateral to the cued as comparedwith the uncued stimulus (*M*= -3.467 %,*SE*= 1.777,*t*(54) = 2.945,*p*= .014,*d*= -0.468,*95%-CI_d_*= [-0.811 -0.125]), whileneither differences between modulations contralateral to the neutral and uncuedside (*M*= 2.601 %,*SE*= 1.777,*t*(54) = 2.209,*p*= .063,*d*= 0.351,*95%-CI_d_*= [0.018 0.683]) nor contralateral to the cued and neutral side(*M*= -0.867 %,*SE*= 1.777,*t*(54) = 0.736,*p*= .465,*d*= -0.107,*95%-CI_d_*= [-0.437 0.437203]) were significant. In addition, planned contrastsrevealed pre- to postcue modulations not to be different from zero(contralateral to the cued side:*M*= -2.297 %,*SE*= 1.401,*t*(44.085) =1.639,*p*= .325,*d*= -0.310,*95%-CI_d_*= [-0.700 -0.080];contralateral to neutrally cued side:*M*= -1.431 %,*SE*= 1.401,*t*(44.085) =1.021,*p*= .626,*d*= -0.193,*95%-CI_d_*= [-0.577 0.192];contralateral to uncued side:*M*= 1.170 %,*SE*= 1.401,*t*(44.085) =0.835,*p*= .626,*d*= 0.158,*95%-CI_d_*= [-0.226 0.541]).

Motor alpha-band activity recorded at two lateralized electrodes over motorregions showed cue-related modulations (seeFig.5) as revealed by a main effect of the factor cue in the repeatedmeasures ANOVA (*F*(1.981,53.497) = 9.892,*p*< .001, η_g_² =0.112).

While motor alpha-band activity contralateral to uncued as well as neutrally cuedtargets was not modulated in amplitude after the cue, as evaluated byHolm-corrected planned comparisons of the marginal means of the ANOVA model(contralateral to uncued stimulus:*M*= 0.783 %,*SE*= 1.374,*t*(55.023) =0.570,*p*= .999,*d*= 0.108,*95%-CI_d_*= [-0.272 0.488];contralateral to neutrally cued stimulus:*M*= -0.761 %,*SE*= 1.374,*t*(55.023) =0.554,*p*= .999,*d*= -0.105,*95%-CI_d_*= [-0.484 0.275]), motoralpha-band amplitude contralateral to the cued target decreased after the cue(*M*= -5.185 %,*SE*= 1.374,*t*(55.023) = 3.775,*p*= .001,*d*= 0.713,*95%-CI_d_*= [-1.139 -0.288]). Accordingly, differences between motor alphamodulations contralateral to cued and uncued (*M*= 5.968%,*SE*= 1.393,*t*(54) = 4.285,*p*< .001,*d*= 0.821,*95%-CI_d_*= [0.376 1.266]) as well ascued and neutrally cued target (*M*= 4.424 %,*SE*= 1.393,*t*(54) = 3.177,*p*= .005,*d*= 0.609,*95%-CI_d_*= [0.190 1.027]) weresignificantly different, while the differences contralateral to neutrally cuedand uncued target did not differ (*M*= 1.543 %,*SE*= 1.393,*t*(54) = 1.108,*p*= .273,*d*= 0.212,*95%-CI_d_*= [-0.176 0.601]).

### 3.5 Relationship between reaction times and cue validity and postcue electrophysiological measures

Unsurprisingly, across all models, the marginal effects of the factor cuevalidity were comparable: models predicted reaction times to be shortest intrials with a valid cue and longest in trials with an invalid cue with reactiontimes for neutrally cued targets falling in between (seeFig. 6). In addition, for all pairwise comparisons, the 95%highest posterior density (HPD) of the posterior distributions of all modeldraws were different from zero and, thus, revealed reaction time differencesbetween all cue validity levels (seeFig. 6andTable 3).

**Fig. 6.**
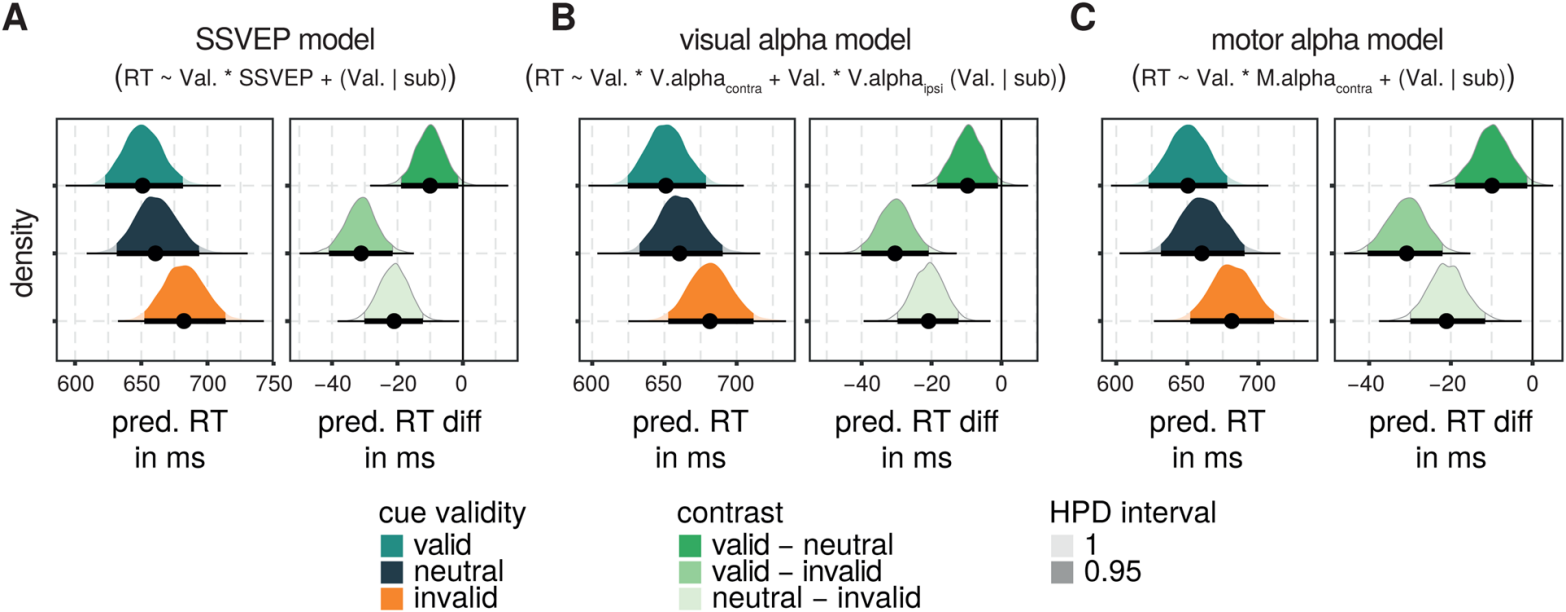
Posterior distributions of effects of cue validity for postcue models.(A) Posterior density distribution of median reaction times predictedfor different levels of the factor cue validity for all model draws ofthe model containing postcue SSVEP amplitudes as a potential additionalpredictor is plotted on the left. On the right, the posterior densitydistributions of predicted median reaction time differences for pairedcontrasts between all factor levels are plotted. The 95% highestposterior density (HPD) interval is indicated by saturated colors andthe bold horizontal line. Median predicted reactions times and reactiontime differences are indexed by a black circle. (B) As in (A) butresults are plotted for the model containing postcue visual alpha-bandamplitudes. (C) As in (A) but results are plotted for the modelcontaining postcue motor alpha-band amplitudes.

**Table 3.** Marginal posterior summaries for cue validity contrasts for all postcuemodels.

Model	Contrast	Predicted reaction time difference in ms		
		Marginal mean	2.5 % HPD	97.5 % HPD
SSVEP model RT ~ Val. * SSVEP + (Val. | sub)	Valid—neutral	-10.034	-18.856	-1.369
	Valid—invalid	-31.110	-40.501	-21.172
	Neutral—invalid	-21.039	-30.406	-12.711
Visual alpha model RT ~ Val. * V.alpha _contra_ + Val. * V.alpha _ipsi_ (Val. | sub)	Valid—neutral	-9.615	-17.975	-0.628
	Valid—invalid	-30.466	-40.172	-21.087
	Neutral—invalid	-20.840	-29.385	-12.084
Motor alpha model RT ~ Val. * M.alpha _contra_ + (Val. | sub)	Valid—neutral	-9.877	-18.786	-1.192
	Valid—invalid	-30.797	-40.425	-22.155
	Neutral—invalid	-21.046	-29.723	-11.461

**Fig. 7.**
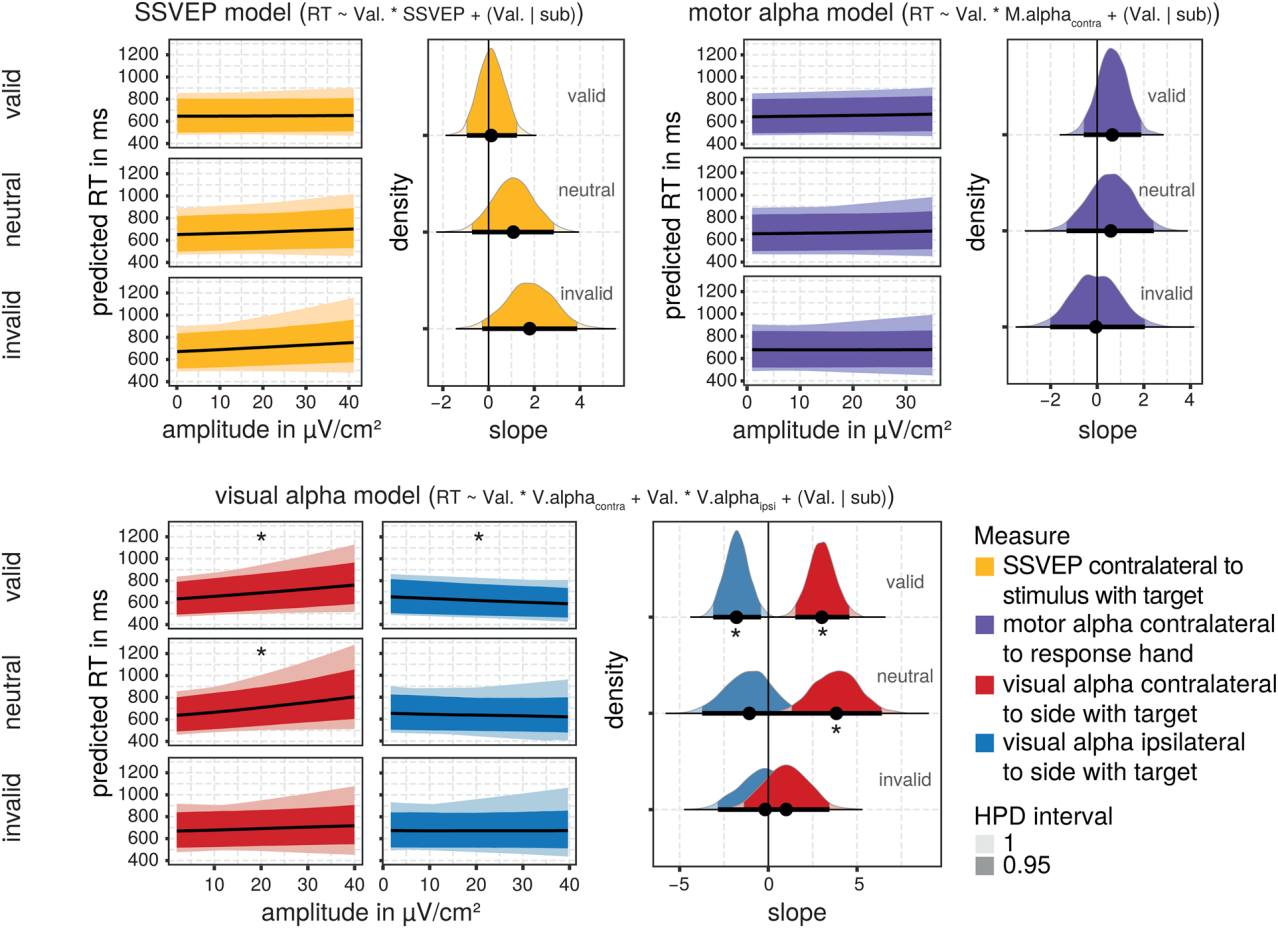
Posterior distributions of effects of postcue electrophysiologicalmeasures on reaction times. The predicted relationship between theamplitude of postcue electrophysiological measures and reaction times isdisplayed separately for each cue validity condition and measure. The95% highest posterior density (HPD) interval for the predictedrelationship across model draws is depicted by the ribbon of thesaturated color. The median of the distribution is indexed by the blackline. The respective density distribution of predicted slope values isplotted on the right side with the 95% HDP intervals depicted by thesaturated colors and bold horizontal lines at the bottom of eachdistribution. Median slopes are indexed by a black circle. *marks the distributions for which all predicted slopes within the 95%HDP interval are different from zero.

Figure 7depicts the effects of therespective electrophysiological measure upon reaction times. Neither for SSVEPsnor for motor alpha-band activity there was a consistent relationship capturedin the respective models across model draws. Here the 95% HPD intervals ofposterior distributions of the predicted slopes included zero, suggesting thatthere is no substantial and consistent positive (or negative) relationshipbetween these neural measures and reaction times captured across model draws(seeTable 4).

**Table 4.** Marginal posterior summaries for predicted slopes of the relationshipbetween the amplitude of postcue neural measures and RTs for cuevalidity conditions, respectively.

Neural measure	Cue validity	Predicted slope		
		Median	2.5 % HPD	97.5 % HPD
**SSVEP**	Valid	0.115	-0.958	1.226
	Neutral	1.082	-0.741	2.805
	Invalid	1.802	-0.330	3.798
***Visual alpha contralateral***	***Valid***	***3.015***	***1.490***	***4.524***
	***Neutral***	***3.842***	***1.287***	***6.350***
	Invalid	1.015	-1.318	3.477
***Visual alpha ipsilateral***	***Valid***	***-1.785***	***-3.127***	***-0.451***
	Neutral	-1.076	-3.960	1.296
	Invalid	-0.192	-2.923	2.538
**Motor alpha contralateral**	Valid	0.649	-0.571	1.891
	Neutral	0.579	-1.197	2.513
	Invalid	-0.058	-2.008	2.052

Note: Substantial marginal effects, that is, for which all slopes inthe 95% highest posterior density (HPD) interval are different fromzero, are in bold italics.

For postcue visual alpha-band amplitudes at electrodes contra- and ipsilateral tothe stimulus with the target, consistent relationships across the model drawswere found. Visual alpha-band amplitudes recorded contralateral to the upcomingtarget were positively correlated with reaction times to target stimuli inneutral and valid, but not invalid trials. The lower the contralateralalpha-amplitudes in the cue–target interval after the valid and neutralcue, the faster the participants responded. For visual ipsilateral alpha-bandactivity, the relation was the opposite for trials with a valid cue. Here higherpostcue amplitudes were related to faster responses. For trials with invalid orneutral cues, no consistent relationship was found.

How well does the visual alpha-band model predict single-trial reaction times?For this model, a Bayesian$R^{2}$value of 0.1917 was extracted. In comparison, a model containing only cuevalidity as a predictor (and no neural measure), that is, the typical Posnereffect, yielded a Bayesian$R^{2}$value of 0.1892, and a mere intercept model for which RT measures were modeledto differ only across participants yielded a Bayesian$R^{2}$value of 0.1801. While the most complex model indeed explained the RT variancethe best, most of the actual variance in the RT data (80.83 %) is not explainedat all.

### 3.6 Relationship between reaction times and cue validity and pretarget electrophysiological measures

In a second step, we looked into the relationship between neurophysiologicalmeasures right before the presentation of a target and single-trial reactiontimes for correct responses. This was based on the idea that potentialfluctuations of neurophysiological measures right before the stimuluspresentation may be more relevant for the processing of and reaction to thebehaviorally relevant target.

In these models, again, the same general relationship between cue validity typeand response times was captured, mirroring the results of the postcue models:participants responded faster for validly cued and much slower for invalidlycued targets with neutrally cued targets falling in between. As above, this wasrevealed by the fact that for all pairwise comparisons between cue validitylevels, the 95% HPD of the posterior distributions of the predicted reactiontime differences did not include zero and thus revealed substantial reactiontime differences (seeTable 5).

**Table 5.** Marginal posterior summaries for cue validity contrasts for all pretargetmodels.

Model	Contrast	Predicted reaction time difference in ms		
		Marginal mean	2.5 % HPD	97.5 % HPD
SSVEP model RT ~ Val. * SSVEP + (Val. | sub)	Valid—neutral	-9.864	-19.260	-1.254
	Valid—invalid	-31.041	-40.518	-21.552
	Neutral—invalid	-21.101	-30.008	-12.356
Visual alpha model RT ~ Val. * V.alpha _contra_ + Val. * V.alpha _ipsi_ (Val. | sub)	Valid—neutral	-9.757	-18.127	-0.717
	Valid—invalid	-30.618	-40.185	-21.119
	Neutral—invalid	-20.887	-29.670	-12.424
Motor alpha model RT ~ Val. * M.alpha _contra_ + (Val. | sub)	Valid—neutral	-9.478	-17.874	-0.911
	Valid—invalid	-30.548	-39.704	-21.126
	Neutral—invalid	-21.059	-29.752	-12.368

**Fig. 8.**
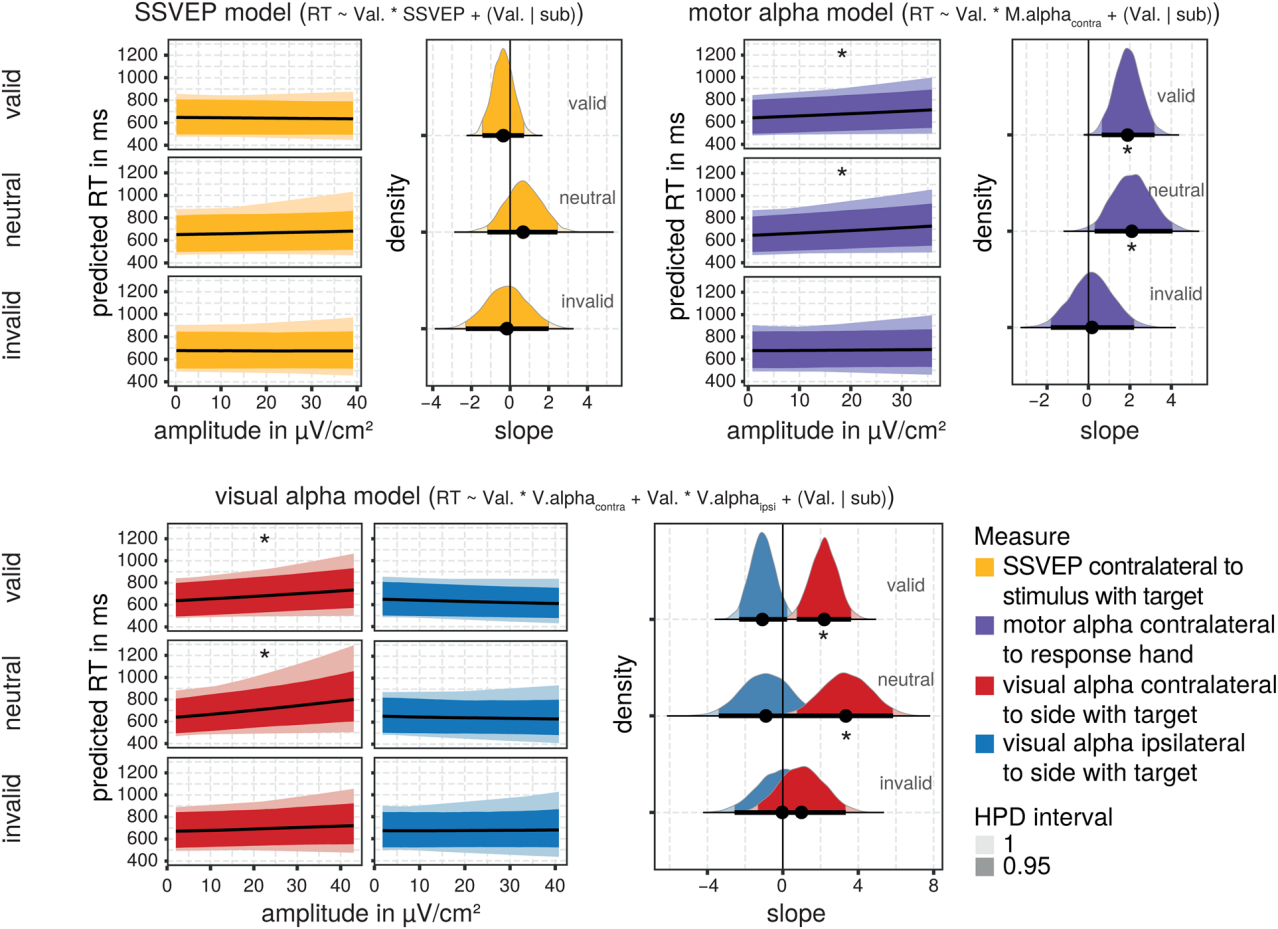
Posterior distributions of effects of pretarget electrophysiologicalmeasures on reaction times. The predicted relationship between theamplitude of postcue electrophysiological measures and reaction times isdisplayed separately for each cue validity condition and measure. The95% highest posterior density (HPD) interval for the predictedrelationship across model draws is depicted by the ribbon of thesaturated color. The median of the distribution is indexed by the blackline. The respective density distribution of predicted slope values isplotted on the right side with the 95% HDP intervals depicted by thesaturated colors and bold horizontal lines at the bottom of eachdistribution. Median slopes are indexed by a black circle. *marks the distributions for which all predicted slopes within the 95%HDP interval are different from zero.

Figure 8andTable 6show the results of the predicted relationshipbetween the respective electrophysiological measures and reaction times in thiswindow before target onset. We found some similarities to the findings for thepostcue neural measures. Again, single-trial SSVEP amplitudes did not predictreaction times across all model draws in a consistent manner. As for the postcuetime window, visual alpha-band amplitudes were related to response times.However, in contrast to the results of the postcue time window, effects wereonly found for amplitudes at sites contralateral to the target side in this timewindow: For trials with a valid and neutral (but not invalid) cue, lowerpretarget contralateral amplitudes were associated with faster response times.Pretarget ipsilateral visual alpha amplitudes were not associated with theactual response time. In contrast to the earlier postcue time window, motoralpha-band amplitudes recorded right before the target presentation were also aconsistent predictor of actual reaction times. As for visual alpha-bandactivity, lower pretarget motor alpha-band amplitudes contralateral to theresponse hand were associated with faster responses for trials with valid orneutral but not invalid cues.

**Table 6.** Marginal posterior summaries for predicted slopes of the relationshipbetween pretarget neural measures’ amplitude and RTs separatelyfor the cue validity conditions.

Neural measure	Cue validity	Predicted slope		
		Median	2.5 % HPD	97.5 % HPD
**SSVEP**	Valid	-0.170	-2.361	1.912
	Neutral	0.676	-1.088	2.493
	Invalid	-0.170	-2.361	1.912
***Visual alpha contralateral***	***Valid***	***2.198***	***0.708***	***3.552***
	***Neutral***	***3.336***	***0.872***	***5.942***
	Invalid	0.997	-1.367	3.280
**Visual alpha ipsilateral**	Valid	-1.079	-2.307	0.235
	Neutral	-0.891	-3.283	1.785
	Invalid	-0.020	-2.563	2.507
***Motor alpha contralateral***	***Valid***	1.890	0.608	3.127
	***Neutral***	2.098	0.385	4.126
	Invalid	0.165	-1.840	2.180

Note: Substantial marginal effects, that is for which all slopes inthe 95% highest posterior density (HPD) interval are different fromzero, are in bold italics.

## 4 Discussion

We examined the interaction between attention, different electrophysiological markersof visuospatial attention, and reaction times in a spatial probabilistic cueingtask. As in previous work and as expected, we found that SSVEP, visual alpha-band,and motor alpha-band activity were modulated following a spatial attentional cue:After the onset of the cue, SSVEP amplitudes were higher for the cued and neutrallycued as compared with the uncued stimulus. Visual alpha-band amplitudes were lowercontralateral to the cued as compared with uncued side, and motor alpha-bandactivity was lower contralateral to the cued stimulus-response hand as compared withthe neutrally cued and uncued response hand. The validity of the cue affected thecorrectness of responses as well as reaction times to targets, with participantsresponding more accurately and faster to validly cued targets compared withresponses in the other conditions. Beyond this cue validity-dependent modulation ofresponse times, we found that trial-by-trial fluctuations of alpha-band activityseem to predict reaction times in the valid and neutral cue condition. In thecue–target interval, lower visual alpha-band amplitudes contralateral to thestimulus with the target were associated with shorter response times after a validor neutral cue. Higher ipsilateral visual alpha amplitudes predicted faster reactiontimes for valid cues in this interval. Ipsilateral alpha was now no longerpredictive of reaction times in the window before target onset, but lower visualalpha-band amplitudes at contralateral electrodes continued to be associated withfaster reaction times in that time window. In addition, lower motor alpha amplitudescontralateral to the response hand were associated with faster reaction times inthis window following valid and neutral cues.

Our findings are well in line with previous reports on behavioral effects in spatialcueing paradigms: for validly cued events, behavioral advantages were usually found,such as faster reaction times, fewer errors, and more correct responses, while forinvalidly cued events, behavioral costs were prominent, leading to slower and lessaccurate responses (seeCarrasco, 2011;Posner, 1980). In seminal studies toinvestigate neural responses in such spatial cueing designs, it was commonly foundthat early components of the visual event-related potential (VEP) such as the N1 orP1 component were modulated in amplitude with attention, resulting in higheramplitudes when a stimulus occurred at the cued, compared with when it occurred atthe uncued side (Hillyard & Anllo-Vento,1998;Hillyard et al., 1998;Luck, 1995;Luck et al., 1997,^2000^;Marzecová et al., 2018;Slagter et al., 2016). These amplitude differences wereinterpreted as a top–down guided sensory gain control mechanism of attention(Hillyard et al., 1998). SSVEP amplitudemodulations had been linked to changes in sensory gain as well (Di Russo et al., 2001;Kim et al., 2007;Müller &Hillyard, 2000), given that SSVEP amplitudes are enhanced for an attendedstimulus compared with when this stimulus needed to be ignored (cf.Gundlach et al., 2020;Müller & Hillyard, 2000;Müller, Teder-Sälejärvi, et al., 1998;Walter et al., 2014). Interestingly, amplitudedecreases for the unattended stimulus relative to a precue baseline were usually notfound (Gundlach et al., 2020;Müller & Hillyard, 2000;Müller, Teder-Sälejärvi, et al.,1998;Walter et al., 2014),indicating that the to-be-ignored stimulus was not further suppressed by spatialattention.

We replicated this pattern in the present study. Of interest is the fact that wefound no differences in SSVEP amplitude gain for trials in which the cue pointed toone side compared with a neutral cue, pointing to both sides. In case of a split ofattentional resources to both sides, one would expect lower amplitudes in theneutral cue condition compared with when all resources were shifted to one side ofthe screen, if attention relied on one common resource pool. A possible explanationfor the fact, that this pattern was not found, offers the so-called differenthemifield advantage that hypothesizes independent pools of attentional resources forboth cortical hemispheres (Alvarez & Cavanagh,2005;Mayo & Maunsell,2016;Störmer et al., 2014;Walter et al., 2014). In this scenario,attentional resources are not shifted between cortical hemispheres (because eachhemisphere has its own), resulting in similar SSVEP amplitudes in situations whenone side needs to be attended or both sides are attended simultaneously.

However, although the SSVEP amplitude pattern for time domain averaged SSVEPsobviously represents sensory gain amplification initiated by the cue, single-trialSSVEP amplitude fluctuations were not related to reaction times in any condition. Ofnote, this finding was mirrored in an exploratory control analysis (seeSupplementary Material).Here visual alpha-band amplitudes contra and ipsilateral to the side with theupcoming target and SSVEP amplitudes contralateral to the stimulus with the targetwere modeled together, mirroring the same relation for visual alpha-band amplitudesas well as no consistent relationship between SSVEP amplitudes and reaction times.As this finding was unexpected and points to a missing effect of SSVEPs, we have noclear explanation why this was the case and may offer only post hoc hypotheses. Onereason might be related to the SNR in single-trial SSVEP extraction. Despite thefact that we employed denoising techniques such as RESS to enhance SSVEP-SNR,specifically suited for single-trial analysis regimes (Cohen & Gulbinaite, 2017), it does not preventnonphase-locked signals from ongoing neural oscillatory activity in the presentSSVEP frequency range (such as beta-band activity) to actually contribute to theSSVEP signals. In a common trial-averaging approach to study SSVEP effects, thesepossible nonphase-locked contributions are averaged out (or are at leastsignificantly reduced) when time domain signals are averaged across trials first,before signals are transferred into frequency domain. Thus, trial-averaged SSVEPsmore robustly reflect sensory gain mechanisms of pure stimulus evoked activity.Single-trial frequency domain data, on the other hand, may be more contaminated bynonstimulus evoked activity, thus, potentially masking the relationship betweensensory gain modulations and behavior. Yet, these single-trial SSVEP amplitudes dodepict the general expected cue-related attentional modulation. One may thushypothesize that SSVEPs may not necessarily and only under certain circumstances bepredictive of behavior. Attention-related behavioral benefits could arise from theimplementation of attention at (hierarchically) different stages (Buschman, 2015;Hommel etal., 2019;Luo & Maunsell,2015;Maunsell, 2015). Signaturesof attention selection at different stages may be unrelated (see for the missingrelationship between SSVEPs and alpha-band activity:Antonov et al., 2020;Gundlach et al.,2020;Nuttall et al., 2022;Zhigalov & Jensen, 2020), may depictdifferent modulation profiles for different cueing conditions as in our data (i.e.,comparing the effect of cue validity on SSVEPs, and visual or motor alpha-bandamplitudes), and may be differently relevant for single-trial behavioral responses(Wyart et al., 2015).

Parieto-occipital alpha-band activity in the cue–target interval showed theexpected attention-related modulatory pattern as well: following the attentionalcue, parieto-occipital alpha-band activity was lateralized and was lowercontralateral to the cued as compared with the uncued side with amplitudes inneutrally cued trials falling somewhere in between. Such a pattern has long beenreported in the visual domain (Antonov et al.,2020;Bauer et al., 2014;Bollimunta et al., 2011;Capotosto et al., 2009;Foster et al., 2017;Gould et al.,2011;Gundlach et al., 2020;Händel et al., 2011;Keefe & Störmer, 2021;Kelly et al., 2006;Liuet al., 2022;Lobier et al., 2018;Samaha et al., 2016;Sauseng et al., 2005;Siegel et al., 2008;Slagter et al.,2016;Sokoliuk et al., 2019;Thut et al., 2006;Voytek et al., 2017;Worden et al., 2000;Zhigalov &Jensen, 2020), and also in the somatosensory domain (Anderson & Ding, 2011;Forschack et al., 2017;Haegens, Händel, et al., 2011;Haegens, Nácher, et al., 2011;Haegens et al., 2012;Jones et al.,2010;van Ede et al., 2011;Wiesman & Wilson, 2020) and auditorydomain (Boudewyn & Carter, 2018;Deng et al., 2019,^2020^;Frey et al.,2014;Strauß et al., 2014;Tune et al., 2018,^2021^;Wöstmann etal., 2016,^2019^). These findingsled to the idea that alpha-band activity represents a neural marker for altering theneural information flow, with some accounts focusing on a potential functional (evenactive) inhibitory role in stimulus processing and attention (Foxe & Snyder, 2011;Jensen & Mazaheri, 2010;Klimesch et al., 2007;Mathewson et al., 2011). However, a recent review that evaluated theevidence in favor of a general (active or causal) inhibitory role of alpha-bandactivity during attention found mixed results for the relationship betweenprestimulus alpha amplitudes and changes in stimulus-evoked neural activity (Morrow et al., 2023).

While our results for valid and, to some extent for neutral trials, in thecue–target interval can be seen as supportive for the proposed hypothesisthat alpha-band modulations are predictive for behavioral responses duringattention, for invalid trials this was not the case. As we outlined in theIntroduction section, if alpha-band activity was generally relevant for attentionalselection, it should be predictive of fast responses in the invalid condition aswell. Here, one would assume that in invalid trials with relatively faster reactiontimes to the uncued target stimulus, the alpha-lateralization pattern should bereversed during the cue–target interval as compared with the prototypicalpattern. Such trial-by-trial reversals of the alpha lateralization for the same cueare indeed measurable and can be a consequence of fluctuations, as we have found inour previous study (Gundlach et al., 2020).Other studies also reported of fluctuations in attention across time (Adam & deBettencourt, 2019;Esterman & Rothlein, 2019;Guilford, 1927;Rosenberg et al., 2015;Yamashita etal., 2021) and the actual alternation of attention between stimuli (Fiebelkorn & Kastner, 2019;Helfrich et al., 2018;VanRullen, 2018). If alpha-band amplitudes played acentral role for attention, one would expect that alpha-band fluctuations depictattentional fluctuations. However, neither cue–target nor pretarget intervalalpha fluctuations predicted reaction times for invalid trials. This was also truefor motor alpha-band activity in these trials. Thus, alpha-band amplitudes seemunrelated to behaviorally relevant fluctuations of attention.

Interestingly, in a probabilistic cueing design,Händel et al. (2011)found that alpha lateralization in thecue–target interval was, in contrast to our findings, correlated withbehavioral responses for trials with an invalid cue,but—surprisingly—not for valid trials. They explained their findingswith competitive interactions that needed to be resolved for the invalid but not forthe valid condition. While this interpretation is open for discussion, it isimportant to know that they used perceptual threshold measures as behavioralresponses that may account for the differing results of their and the current study.In their study, subjects needed to indicate the direction of coherent motion eventsafter 2 seconds interval between the presentation of a target display, consisting oftwo potentially task-relevant and competing stimuli, and the postcued, requiredresponse to one of the previously presented stimuli. While alpha-band activity waslateralized in the cue–target interval, there was no lateralization in thetarget–response time window. Obviously, our discrimination task differs, aswe used immediate reaction times to a single target as the behavioral measurewithout any delay between target and response and without any actual distractorsbeing presented at all. In addition, results stemmed from the correlation betweenalpha-band amplitudes and behavior across subjects and not from trial-by-trialvariations of alpha-band amplitudes, as analyzed in our study. Whether the taskdifferences are responsible for the contradictory results between the two studies isan open question, but the idea of a general active inhibitory role of alpha-bandactivity in stimulus processing may not be warranted.

Motor alpha-band amplitudes recorded contralateral to the cued side, that is, theresponse hand, were decreased compared with ipsilateral central electrodes. Intrials with a neutral cue, there were no substantial modulation of motor alpha-bandactivity. For valid and neutral trials, these lower amplitudes measured in apretarget interval were predictive of faster reaction times, implying that low motoralpha amplitudes may be associated with facilitated and faster responses. Recentwork found Transcranial-Magnetic-Stimulation (TMS) evoked motor-evoked-potential(MEP) pulses to be modulated by the amplitude (and phase) of ongoing motoralpha/mu-alpha-band activity (Hussain et al.,2018;Karabanov et al., 2021;Ogata et al., 2019;Thies et al., 2018; but seeZrenner et al., 2022). Lower amplitude levels forinstance led to larger MEPs. TMS protocols illustrating inhibitory neural processesfound measures not to be modulated by motor alpha-band activity (Bergmann et al., 2019). These findings point toward thefacilitatory role of alpha-band activity in the motor cortex representing thegeneral excitatory state of the system. Our findings are in line with this idea ofneural excitability, and the decreased alpha-band levels contralateral to the cuedside may point toward preparatory processes in the motor cortex associated with thecued response.

The interpretation of motor alpha-band activity as a neural signature of neuralexcitability in motor cortex to prepare motor execution is well in line with recentsuggestions that relative alpha-band fluctuations seem to be associated with generalchanges in excitability of neural populations: higher alpha-band amplitudes seem toindex lower excitability levels (Iemi et al.,2022;Samaha et al., 2020;Van Diepen et al., 2019). In our experiment,these behaviorally relevant excitability changes depicted by alpha-band activitywere relevant for trials with a valid or a neutral cue only, and were not found fortrials with an invalid cue. Does this mean that alpha-band activity is not relatedto changes in attentional dynamics, attentional reallocation processes, or resourceallocation at cued and uncued locations (Carrasco,2011;Eckstein et al., 2002;Macaluso & Doricchi, 2013;Posner, 1980)? This is hard to answer solelybased on our results. During the cue–target interval, each subjects’best strategy for fast responses was to shift attention to the cued side, as thetarget appeared three times more often at the cued side, or split attention to bothsides following a neutral cue.

If alpha-band activity was relevant for instantiating the behaviorally relevantattentional selection, one could assume alpha-band fluctuations contralateral to astimulus to depict fluctuations in the attentional selection of this stimulus.Irrespective of the actual cue validity, alpha-band amplitudes should depict thegraded attentional selection of the stimulus and lower amplitudes be indicative offaster responses. This pattern was, however, not found for invalid trials.Specifically, for invalid trials, the presentation of the target on the uncued sidewould require a reallocation of attention and shifting of attentional resources inorder to respond to the target. In invalid trials, alpha-band amplitudes seem not toindex the initial attentional selection of the uncued side or the fidelity of theattentional reallocation process. Further, in these invalid trials, theattention-related modulation of cortical excitability could only occur after thepresentation of the target and the consequent shift of attention. Therefore,alpha-band amplitude fluctuations seem to be behaviorally relevant only when nospatial attentional reallocation is required and may index behaviorally excitabilitychanges only as a consequence of top–down driven attentional selection andresource allocation. As seen in previous work, the behavioral relevance ofalpha-band modulations may be related to the confidence in selecting the cuedlocation (Pilipenko & Samaha, 2024;Samaha et al., 2017,^2020^), This idea is supported by the fact that after thepresentation of the target and after the reallocation of attention in invalidtrials, we found for all targets in all conditions a similar behaviorally relevantmotor–alpha desynchronization contralateral to the response hand, with loweramplitude values indicating higher excitability of the motor system, leading tofaster responses (seeSupplementary Material). If participants followed a different strategyin the sense of “preparatory” alternations of attention between thetwo stimuli (because subjects know that there are some invalid trials as well) andalpha-band fluctuations depicted these behaviorally relevant fluctuations in theactual attentional selection, one would expect alpha-band amplitudes to bebehaviorally relevant in invalid trials as well. As we did not find this pattern,this underscores the idea that alpha-band modulations are the consequence oftop–down activity in the present experiment, and it is unlikely thatspontaneous alpha-band fluctuations represent a mechanism that actively alternatestop–down guided allocation of attentional resources.

This interpretation is further supported by the present findings (seeSupplementary Material“Relationship of Post-cue Electrophysiological Measures andAttention”), which show that single-trial visual alpha-band amplitudes arenot consistently predictive of SSVEP amplitudes. Additionally, several recentstudies have reported that fluctuations in the alpha band do not influence SSVEPamplitudes, and therefore, do not affect early visual stimulus processing (Antonov et al., 2020;Gundlach et al., 2020;Nuttall et al., 2022;Zhigalov &Jensen, 2020). As mentioned above, SSVEPs robustly representtop–down guided neural sensory gain control in early visual cortex (Di Russo et al., 2001;Kim et al., 2007;Müller & Hillyard, 2000;Müller, Picton, et al., 1998;Müller, Teder-Sälejärvi, et al., 1998) (see alsocurrent study), this renders the view very unlikely that attentional alteration ofstimulus processing in early visual cortex is mechanistically implemented by visualalpha-band activity. To what extent visual alpha-band activity reflects a gatingmechanism at later processing stages (Peylo et al.,2021;Zhigalov & Jensen,2020) that operates independently from stimulus processing at earlyvisual stages and may index the readout of behaviorally relevant visual information(Chaumon & Busch, 2014) is still amatter of debate. Our data are in line with the proposed idea of a gating mechanismat later stages, but would suggest this gating to be a consequence of the allocationof attention. Of interest, in contrast to the here reported lack of a relationshipbetween alpha-band activity and early sensory processing in recent work byIemi et al. (2019), alpha-band fluctuationswere in fact predictive of early sensory processing, as the early visual C1component evoked by transiently presented wedges of checkerboard patterns wasmodulated by prestimulus alpha-band amplitudes. One crucial difference to our studywas that in their study, effects of alpha-band amplitudes were studied underconstant attentional allocation, that is, effects of lateralized alpha-bandamplitudes were measured while participants were engaged in a central taskthroughout the experiment, while in our study spatial attentional selection of oneside was manipulated on a trial-by-trial level. In addition, in our study,alpha-band amplitude measures were derived during constant visual stimulation, whileforIemi et al. (2019), signals fromprestimulation periods without visual stimulation served as predictors. Whetherpotentially different fluctuations of alpha-band amplitudes were captured in bothexperiments, leading to these differential effects, will need to be addressed infuture work.

Finally, some caution must also be mentioned when addressing the behavioral variancefound in such designs. The classical Posner effect in this study resulted in averagereaction time differences of 29.641 ms between valid and invalid trials. At the sametime, there is substantial variation in response times across participants (grandmean average reaction time = 663.872 ms; SD of mean reaction times acrossparticipants = 64.899). RTs vary even more within each subject (seeFig. 2A) with an average SD of 133.509 ms. Theactual Posner effect accounted only for a small part of variation within the data.In fact, extracting Bayes-R² values for the models fitted to the single-trialreaction time data revealed that only 18.010% of variance was explained by generalreaction time offsets and, thus, general differences between participants. Around 1%additional variance was explained by the factor cue validity, that is, the classicalPosner effect (18.922%) and less than 1% was additionally explained by postcuealpha-band activity at contralateral electrodes (resulting in overall 19.168% of theexplained variance). While effects such as the classical Posner effect have beenfound and replicated in various experiments and publications, about 80% ofsingle-trial variance was not explained with our models. Recent work, however,points toward the functional significance of variance in biological systems toactually allow flexible, precise, and robust propagation of information andultimately behavioral responses (Ecker et al.,2016;McGinley et al., 2015;Rowland et al., 2023;Schölvinck et al., 2015;Vinck et al., 2015;Waschke et al., 2021). Future efforts need to be made to find,disentangle, and characterize potential additional determinants of neural andbehavioral variability and the role and profile of noise for neuralcomputations.

Our analyses were limited to time windows which in this and previous studies revealedextensive modulations of neural activity under sustained attention. We addressedwhether trial-by-trial fluctuations in neural measures for a postcue window ofsustained attention are behaviorally relevant. While we also examined fluctuationson a time scale closer to the actual processing of the target stimulus, neuralmeasures were aggregated across a time window of 1 second in all approaches. It,thus, remains an open question, how dynamics and fluctuations on smaller time scalesmay contribute to behavior. In addition, our design did not include a graded measureof accuracy within each trial. The relevance of trial-by-trial fluctuations ofneural measures related to behavioral accuracy will need to be evaluated in futurestudies.

Furthermore, while our analyses revealed alpha-band activity in both hemispheres tobe relevant and an enhanced alpha-lateralization pattern to be behaviorallybeneficial in valid trials, the functional interpretation may be ambiguous.Alpha-band modulations, typically characterized by a decrease contralateral to thecue and an increase ipsilateral to it, have been associated with enhanced processingof the target stimulus and reduced processing of distracting information(contralateral to the uncued stimulus) (seeCapillaet al., 2014). Yet, both signals may be confounded and driven by abilateral modulation related to the processing of the target stimulus. In fact, inour design, no potentially distracting events were presented at all and a functionalinterpretation with regard to the attenuation of distraction information and/or theenhancement of target information are, therefore, not possible. A distinct design,such as used by Orf et al. (Orf et al.,2023), would be required for disentangling target enhancement and distractorsuppression-related processes.

To summarize, we found modulations of behavior, SSVEP, visual, and motor alpha-bandactivity through top–down guided spatial attention in a probabilistic spatialcueing task. These findings mirror previous results and are extended by evaluatingthe behavioral relevance of the attention-modulated but unrelated neural measures.Here, fluctuations in visual alpha-band activity measured right after the cue andstretching until target presentation as well as fluctuations in motor alpha-bandactivity obtained just before target presentation were linked to response times totarget stimuli in valid and neutral but not invalid trials, which conflicts with theidea of alpha-band fluctuations signify top–down guided allocation ofattentional resources (Gundlach & Forschack,2020). If alpha-band activity would have played an active role intop–down attentional deployment, one would expect larger amplitudescontralateral and/or smaller amplitudes ipsilateral to the cued hemifield to berelated to the faster switching of attention to the target at the uncued side ininvalid trials, and hence faster reaction times. As this was not the case, wequestion the active role of alpha-band fluctuations in stimulus processing in suchdesigns. Instead, and in line with some previous studies, we suggest thatbehaviorally relevant alpha-band modulations are the consequence of top–downguided spatial attention, representing neural excitability in cortical areas thatare activated by the attentional shift. Spontaneous fluctuations of neuralexcitability in these areas have an influence on stimulus processing for the cuedside, but will not overwrite top–down guided shifts of attentional resourcesto one side as long as it is the best strategy to follow the cue for bestperformance.

## Supplementary Material

Supplementary Material

## Data Availability

The data and code used for the analyses and results presented in this study areavailable at:https://osf.io/6ygvm/.

## Footnotes

* The longer epochs were also motivated to allow for Gabor filter-based time courseamplitude analyses that require larger time windows as amplitude values near theboundaries of the time windows are hard to interpret due to edge artifacts.However, the exact time courses of alpha and SSVEP amplitudes are not part ofthe present study and might be reported elsewhere.

## References

[b1] Adam , K. C. S. , & deBettencourt , M. T. ( 2019 ). Fluctuations of attention and working memory . J Cogn , 2 , 33 . 10.5334/joc.70 31440739 PMC6696791

[b2] Adrian , E. D. , & Matthews , B. H. C. ( 1934 ). The berger rhythm: Potential changes from the occipital lobes in man . Brain , 57 , 355 – 385 . 10.1093/brain/57.4.355 20058345

[b3] Alvarez , G. A. , & Cavanagh , P. ( 2005 ). Independent resources for attentional tracking in the left and right visual hemifields . Psychol Sci , 16 , 637 – 643 . 10.1111/j.1467-9280.2005.01587.x 16102067

[b4] Anderson , K. L. , & Ding , M. ( 2011 ). Attentional modulation of the somatosensory mu rhythm . Neuroscience , 180 , 165 – 180 . 10.1016/j.neuroscience.2011.02.004 21310216

[b5] Antonov , P. A. , Chakravarthi , R. , & Andersen , S. K. ( 2020 ). Too little, too late, and in the wrong place: Alpha band activity does not reflect an active mechanism of selective attention . Neuroimage , 219 , 117006 . 10.1016/j.neuroimage.2020.117006 32485307

[b6] Babiloni , C. , Capotosto , P. , Brancucci , A. , Percio Del , C., Petrini , L. , Buttiglione , M. , Cibelli , G. , Romani , G. L. , Rossini , P. M. , & Arendt-Nielsen , L. ( 2008 ). Cortical alpha rhythms are related to the anticipation of sensorimotor interaction between painful stimuli and movements: A high-resolution EEG study . J Pain , 9 , 902 – 911 . 10.1016/j.jpain.2008.05.007 18619907

[b7] Bauer , M. , Stenner , M.-P. , Friston , K. J. , & Dolan , R. J. ( 2014 ). Attentional modulation of alpha/beta and gamma oscillations reflect functionally distinct processes . J Neurosci , 34 , 16117 – 16125 . 10.1523/JNEUROSCI.3474-13.2014 25429152 PMC4244475

[b8] Bergmann , T. O. , Lieb , A. , Zrenner , C. , & Ziemann , U. ( 2019 ). Pulsed facilitation of corticospinal excitability by the sensorimotor μ-alpha rhythm . J Neurosci , 39 , 10034 – 10043 . 10.1523/JNEUROSCI.1730-19.2019 31685655 PMC6978939

[b9] Bollimunta , A. , Mo , J. , Schroeder , C. E. , & Ding , M. ( 2011 ). Neuronal mechanisms and attentional modulation of corticothalamic alpha oscillations . J Neurosci , 31 , 4935 – 4943 . 10.1523/JNEUROSCI.5580-10.2011 21451032 PMC3505610

[b10] Boudewyn , M. A. , & Carter , C. S. ( 2018 ). I must have missed that: Alpha-band oscillations track attention to spoken language . Neuropsychologia , 117 , 148 – 155 . 10.1016/j.neuropsychologia.2018.05.024 29842859

[b11] Boylan , M. R. , Panitz , C. , Tebbe , A.-L. , Vieweg , P. , Forschack , N. , Müller , M. M. , & Keil , A. ( 2023 ). Feature-based attentional amplitude modulations of the steady-state visual evoked potentials reflect Blood Oxygen Level Dependent changes in feature-sensitive visual areas . J Cogn Neurosci , 35 ( 9 ), 1493 – 1507 . 10.1162/jocn_a_02030 37432748

[b12] Brainard , D. H. ( 1997 ). The psychophysics toolbox . Spat Vis , 10 , 433 – 436 . 10.1163/156856897x00357 9176952

[b13] Brinkman , L. , Stolk , A. , Dijkerman , H. C. , Lange de , P. F. , & Toni , I. ( 2014 ). Distinct roles for alpha- and beta-band oscillations during mental simulation of goal-directed actions . J Neurosci , 34 , 14783 – 14792 . 10.1523/JNEUROSCI.2039-14.2014 25355230 PMC4212072

[b14] Brinkman , L. , Stolk , A. , Marshall , T. R. , Esterer , S. , Sharp , P. , Dijkerman , H. C. , Lange de , P. F. , & Toni , I. ( 2016 ). Independent causal contributions of alpha- and beta-band oscillations during movement selection . J Neurosci , 36 , 8726 – 8733 . 10.1523/JNEUROSCI.0868-16.2016 27535917 PMC4987441

[b15] Bürkner , P.-C. ( 2017 ). brms: An R package for Bayesian multilevel models using stan . J Stat Softw , 80 , 1 – 28 . 10.18637/jss.v080.i01

[b16] Buschman , T. J. ( 2015 ). Paying attention to the details of attention . Neuron , 86 , 1111 – 1113 . 10.1016/j.neuron.2015.05.031 26050029

[b17] Buzsáki , G. , Logothetis , N. , & Singer , W. ( 2013 ). Scaling brain size, keeping timing: Evolutionary preservation of brain rhythms . Neuron , 80 , 751 – 764 . 10.1016/j.neuron.2013.10.002 24183025 PMC4009705

[b18] Capilla , A. , Schoffelen , J. M. , Paterson , G. , Thut , G. , & Gross , J. ( 2014 ). Dissociated alpha-band modulations in the dorsal and ventral visual pathways in visuospatial attention and perception . Cereb Cortex , 24 , 550 – 561 . 10.1093/cercor/bhs343 23118197 PMC3888375

[b19] Capotosto , P. , Babiloni , C. , Romani , G. L. , & Corbetta , M. ( 2009 ). Frontoparietal cortex controls spatial attention through modulation of anticipatory alpha rhythms . J Neurosci , 29 , 5863 – 5872 . 10.1523/jneurosci.0539-09.2009 19420253 PMC2692025

[b20] Carrasco , M. ( 2011 ). Visual attention: The past 25 years . Vision Res , 51 , 1484 – 1525 . 10.1016/j.visres.2011.04.012 21549742 PMC3390154

[b21] Chaumon , M. , & Busch , N. ( 2014 ). Prestimulus neural oscillations inhibit visual perception via modulation of response gain . J Cogn Neurosci , 26 ( 11 ), 2514 – 2529 . 10.1162/jocn_a_00653 24742156

[b22] Cohen , M. X. , & Gulbinaite , R. ( 2017 ). Rhythmic entrainment source separation: Optimizing analyses of neural responses to rhythmic sensory stimulation . Neuroimage , 147 , 43 – 56 . 10.1016/j.neuroimage.2016.11.036 27916666

[b23] Cousineau , D. , Brown , S. , & Heathcote , A. ( 2004 ). Fitting distributions using maximum likelihood: Methods and packages . Behav Res Methods Instrum Comput , 36 , 742 – 756 . 10.3758/BF03206555 15641420

[b24] Deiber , M.-P. , Sallard , E. , Ludwig , C. , Ghezzi , C. , Barral , J. , & Ibanez , V. ( 2012 ). EEG alpha activity reflects motor preparation rather than the mode of action selection . Front Integr Neurosci , 6 , 59 . 10.3389/fnint.2012.00059 22912607 PMC3418545

[b25] Delorme , A. , & Makeig , S. ( 2004 ). EEGLAB: An open source toolbox for analysis of single-trial EEG dynamics including independent component analysis . J Neurosci Methods , 134 , 9 – 21 . 10.1016/j.jneumeth.2003.10.009 15102499

[b26] Deng , Y. , Choi , I. , & Shinn-Cunningham , B. ( 2020 ). Topographic specificity of alpha power during auditory spatial attention . Neuroimage , 207 , 116360 . 10.1016/j.neuroimage.2019.116360 31760150 PMC9883080

[b27] Deng , Y. , Reinhart , R. M. , Choi , I. , & Shinn-Cunningham , B. G. ( 2019 ). Causal links between parietal alpha activity and spatial auditory attention . Elife , 8 , e51184 . 10.7554/eLife.51184 31782732 PMC6904218

[b28] Di Russo , F. , Spinelli , D. , & Morrone , M. C. ( 2001 ). Automatic gain control contrast mechanisms are modulated by attention in humans: Evidence from visual evoked potentials . Vision Res , 41 , 2435 – 2447 . 10.1016/s0042-6989(01)00134-1 11483175

[b29] Ecker , A. S. , Denfield , G. H. , Bethge , M. , & Tolias , A. S. ( 2016 ). On the structure of neuronal population activity under fluctuations in attentional state . J Neurosci , 36 , 1775 – 1789 . 10.1523/JNEUROSCI.2044-15.2016 26843656 PMC4737784

[b30] Eckstein , M. P. , Shimozaki , S. S. , & Abbey , C. K. ( 2002 ). The footprints of visual attention in the Posner cueing paradigm revealed by classification images . J Vis , 2 , 25 – 45 . 10.1167/2.1.3 12678595

[b31] Esterman , M. , & Rothlein , D. ( 2019 ). Models of sustained attention . Curr Opin Psychol , 29 , 174 – 180 . 10.1016/j.copsyc.2019.03.005 30986621

[b32] Ferree , T. C. ( 2006 ). Spherical splines and average referencing in scalp electroencephalography . Brain Topogr , 19 , 43 – 52 . 10.1007/s10548-006-0011-0 17019635

[b33] Fiebelkorn , I. C. , & Kastner , S. ( 2019 ). A rhythmic theory of attention . Trends Cogn Sci , 23 , 87 – 101 . 10.1016/j.tics.2018.11.009 30591373 PMC6343831

[b34] Forschack , N. , Nierhaus , T. , Müller , M. M. , & Villringer , A. ( 2017 ). Alpha-band brain oscillations shape the processing of perceptible as well as imperceptible somatosensory stimuli during selective attention . J Neurosci , 37 , 6983 – 6994 . 10.1523/JNEUROSCI.2582-16.2017 28630252 PMC6705724

[b35] Foster , J. J. , Sutterer , D. W. , Serences , J. T. , Vogel , E. K. , & Awh , E. ( 2017 ). Alpha-band oscillations enable spatially and temporally resolved tracking of covert spatial attention . Psychol Sci , 28 , 929 – 941 . 10.1177/0956797617699167 28537480 PMC5675530

[b36] Foxe , J. J. , & Snyder , A. C. ( 2011 ). The role of alpha-band brain oscillations as a sensory suppression mechanism during selective attention . Front Psychol , 2 , 154 . 10.3389/fpsyg.2011.00154 21779269 PMC3132683

[b37] Frey , J. N. , Mainy , N. , Lachaux , J.-P. , Müller , N. , Bertrand , O. , & Weisz , N. ( 2014 ). Selective modulation of auditory cortical alpha activity in an audiovisual spatial attention task . J Neurosci , 34 , 6634 – 6639 . 10.1523/jneurosci.4813-13.2014 24806688 PMC6608137

[b38] Gelman , A. , Goodrich , B. , Gabry , J. , & Vehtari , A. ( 2019 ). R-squared for Bayesian Regression Models . Am Stat , 73 , 307 – 309 . 10.1080/00031305.2018.1549100

[b39] Gould , I. C. , Rushworth , M. F. , & Nobre , A. C. ( 2011 ). Indexing the graded allocation of visuospatial attention using anticipatory alpha oscillations . J Neurophysiol , 105 , 1318 – 1326 . 10.1152/jn.00653.2010 21228304 PMC3074422

[b40] Guilford , J. P. ( 1927 ). “Fluctuations of attention” with weak visual stimuli . Am J Psychol , 38 , 534 – 583 . 10.2307/1414394

[b41] Gulbinaite , R. , Roozendaal , D. H. M. , & VanRullen , R. ( 2019 ). Attention differentially modulates the amplitude of resonance frequencies in the visual cortex . Neuroimage , 203 , 116146 . 10.1016/j.neuroimage.2019.116146 31493535

[b42] Gundlach , C. , & Forschack , N. ( 2020 ). Commentary: Alpha synchrony and the neurofeedback control of spatial attention . Front Neurosci , 14 , 597 . 10.3389/fnins.2020.00597 32612505 PMC7308421

[b43] Gundlach , C. , Moratti , S. , Forschack , N. , & Müller , M. M. ( 2020 ). Spatial attentional selection modulates early visual stimulus processing independently of visual alpha modulations . Cereb Cortex , 30 , 3686 – 3703 . 10.1093/cercor/bhz335 31907512

[b44] Haegens , S. , Barczak , A. , Musacchia , G. , Lipton , M. L. , Mehta , A. D. , Lakatos , P. , & Schroeder , C. E. ( 2015 ). Laminar profile and physiology of the α rhythm in primary visual, auditory, and somatosensory regions of neocortex . J Neurosci , 35 , 14341 – 14352 . 10.1523/JNEUROSCI.0600-15.2015 26490871 PMC4683691

[b45] Haegens , S. , Händel , B. F. , & Jensen , O. ( 2011 ). Top-down controlled alpha band activity in somatosensory areas determines behavioral performance in a discrimination task . J Neurosci , 31 , 5197 – 5204 . 10.1523/JNEUROSCI.5199-10.2011 21471354 PMC6622699

[b46] Haegens , S. , Luther , L. , & Jensen , O. ( 2012 ). Somatosensory anticipatory alpha activity increases to suppress distracting input . J Cogn Neurosci , 24 , 677 – 685 . 10.1162/jocn_a_00164 22066587

[b47] Haegens , S. , Nácher , V. , Luna , R. , Romo , R. , & Jensen , O. , ( 2011 ). α-Oscillations in the monkey sensorimotor network influence discrimination performance by rhythmical inhibition of neuronal spiking . Proc Natl Acad Sci USA , 108 , 19377 – 19382 . 10.1073/pnas.1117190108 22084106 PMC3228466

[b48] Händel , B. F. , Haarmeier , T. , & Jensen , O. ( 2011 ). Alpha oscillations correlate with the successful inhibition of unattended stimuli . J Cogn Neurosci , 23 , 2494 – 2502 . 10.1162/jocn.2010.21557 20681750

[b49] Helfrich , R. F. , Fiebelkorn , I. C. , Szczepanski , S. M. , Lin , J. J. , Parvizi , J. , Knight , R. T. , & Kastner , S. ( 2018 ). Neural mechanisms of sustained attention are rhythmic . Neuron , 99 , 854.e5 – 865.e5 . 10.1016/j.neuron.2018.07.032 30138591 PMC6286091

[b50] Hillyard , S. A. , & Anllo-Vento , L. ( 1998 ). Event-related brain potentials in the study of visual selective attention . Proc Natl Acad Sci USA , 95 , 781 – 787 . 10.1073/pnas.95.3.781 9448241 PMC33798

[b51] Hillyard , S. A. , Hinrichs , H. , Tempelmann , C. , Morgan , S. T. , Hansen , J. C. , Scheich , H. , & Heinze , H. J. ( 1997 ). Combining steady-state visual evoked potentials and f MRI to localize brain activity during selective attention . Hum Brain Mapp , 5 , 287 – 292 . 10.1002/(SICI)1097-0193(1997)5:4<287::AID-HBM14>3.0.CO;2-B 20408230

[b52] Hillyard , S. A. , Vogel , E. K. , & Luck , S. J. ( 1998 ). Sensory gain control (amplification) as a mechanism of selective attention: Electrophysiological and neuroimaging evidence . Philos Trans R Soc Lond B Biol Sci , 353 , 1257 – 1270 . 10.1098/rstb.1998.0281 9770220 PMC1692341

[b53] Holm , S. ( 1979 ). A simple sequentially rejective multiple test procedure . Scand Stat Theory Appl , 6 , 65 – 70 . http://www.jstor.org/stable/4615733

[b54] Hommel , B. , Chapman , C. S. , Cisek , P. , Neyedli , H. F. , Song , J.-H. , & Welsh , T. N. ( 2019 ). No one knows what attention is . Atten Percept Psychophys , 81 ( 7 ), 2288 – 2303 . 10.3758/s13414-019-01846-w 31489566 PMC6848248

[b55] Hussain , S. J. , Claudino , L. , Bönstrup , M. , Norato , G. , Cruciani , G. , Thompson , R. , Zrenner , C. , Ziemann , U. , Buch , E. , & Cohen , L. G. ( 2018 ). Sensorimotor oscillatory phase-power interaction gates resting human corticospinal output . Cereb Cortex , 29 ( 9 ), 3766 – 3777 . 10.1093/cercor/bhy255 PMC6686752 30496352

[b56] Iemi , L. , Busch , N. A. , Laudini , A. , Haegens , S. , Samaha , J. , Villringer , A. , & Nikulin , V. V. ( 2019 ). Multiple mechanisms link prestimulus neural oscillations to sensory responses . Elife , 8 , e43620 . 10.7554/eLife.43620 31188126 PMC6561703

[b57] Iemi , L. , Gwilliams , L. , Samaha , J. , Auksztulewicz , R. , Cycowicz , Y. M. , King , J.-R. , Nikulin , V. V. , Thesen , T. , Doyle , W. , Devinsky , O. , Schroeder , C. E. , Melloni , L. , & Haegens , S. ( 2022 ). Ongoing neural oscillations influence behavior and sensory representations by suppressing neuronal excitability . Neuroimage , 247 , 118746 . 10.1016/j.neuroimage.2021.118746 34875382

[b58] Jensen , O. , & Mazaheri , A. ( 2010 ). Shaping functional architecture by oscillatory alpha activity: Gating by inhibition . Front Hum Neurosci , 5 , 12 . 10.3389/fnhum.2010.00186 PMC2990626 21119777

[b59] Jones , S. R. , Kerr , C. E. , Wan , Q. , Pritchett , D. L. , Hamalainen , M. , & Moore , C. I. ( 2010 ). Cued spatial attention drives functionally relevant modulation of the mu rhythm in primary somatosensory cortex . J Neurosci , 30 , 13760 – 13765 . 10.1523/JNEUROSCI.2969-10.2010 20943916 PMC2970512

[b60] Junghöfer , M. , Elbert , T. , Tucker , D. M. , & Rockstroh , B. ( 2000 ). Statistical control of artifacts in dense array EEG/MEG studies . Psychophysiology , 37 , 523 – 532 . 10.1111/1469-8986.3740523 10934911

[b61] Karabanov , A. N. , Madsen , K. H. , Krohne , L. G. , & Siebner , H. R. ( 2021 ). Does pericentral mu-rhythm “power” corticomotor excitability?—A matter of EEG perspective . Brain Stimul , 14 , 713 – 722 . 10.1016/j.brs.2021.03.017 33848678

[b62] Kayser , J. ( 2009 ). Current source density (CSD) interpolation using spherical splines-CSD Toolbox . New York State Psychiatric Institute: Division of Cognitive Neuroscience . http://psychophysiology.cpmc.columbia.edu/Software/CSDtoolbox

[b63] Kayser , J. , & Tenke , C. E. ( 2006 ). Principal components analysis of Laplacian waveforms as a generic method for identifying ERP generator patterns: II. Adequacy of low-density estimates . Clin Neurophysiol , 117 , 369 – 380 . 10.1016/j.clinph.2005.08.033 16356768

[b64] Keefe , J. M. , & Störmer , V. S. ( 2021 ). Lateralized alpha activity and slow potential shifts over visual cortex track the time course of both endogenous and exogenous orienting of attention . Neuroimage , 225 , 117495 . 10.1016/j.neuroimage.2020.117495 33184032

[b65] Keitel , C. , Keitel , A. , Benwell , C. S. Y. , Daube , C. , Thut , G. , & Gross , J. ( 2019 ). Stimulus-driven brain rhythms within the alpha band: The attentional-modulation conundrum . J Neurosci , 39 , 3119 – 3129 . 10.1523/JNEUROSCI.1633-18.2019 30770401 PMC6468105

[b66] Kelly , S. P. , Lalor , E. C. , Reilly , R. B. , & Foxe , J. J. ( 2006 ). Increases in alpha oscillatory power reflect an active retinotopic mechanism for distracter suppression during sustained visuospatial attention . J Neurophysiol , 95 , 3844 – 3851 . 10.1152/jn.01234.2005 16571739

[b67] Kim , Y. J. , Grabowecky , M. , Paller , K. A. , Muthu , K. , & Suzuki , S. ( 2007 ). Attention induces synchronization-based response gain in steady-state visual evoked potentials . Nat Neurosci , 10 , 117 – 125 . 10.1038/nn1821 17173045

[b68] Kleiner , M. , Brainard , D. , Pelli , D. , Ingling , A. , Murray , R. , & Broussard , C. ( 2007 ). What’s new in Psychtoolbox-3 . Perception , 36 ( 14 ), 1 – 16 . https://journals.sagepub.com/doi/pdf/10.1177/03010066070360S101

[b69] Klimesch , W. , Sauseng , P. , & Hanslmayr , S. ( 2007 ). EEG alpha oscillations: The inhibition-timing hypothesis . Brain Res Rev , 53 , 63 – 88 . 10.1016/j.brainresrev.2006.06.003 16887192

[b70] Labecki , M. , Kus , R. , Brzozowska , A. , Stacewicz , T. , Bhattacharya , B. S. , & Suffczynski , P. ( 2016 ). Nonlinear origin of SSVEP Spectra-A combined experimental and modeling study . Front Comput Neurosci , 10 , 129 . 10.3389/fncom.2016.00129 28082888 PMC5187367

[b71] Lenth , R. V. ( 2023 ). emmeans: Estimated Marginal Means, aka Least-Squares Means . 10.32614/CRAN.package.emmeans

[b72] Liu , B. , Nobre , A. C. , & Ede van , F . ( 2022 ). Functional but not obligatory link between microsaccades and neural modulation by covert spatial attention . Nat Commun , 13 , 3503 . 10.1038/s41467-022-31217-3 35715471 PMC9205986

[b73] Lobier , M. , Palva , J. M. , & Palva , S. ( 2018 ). High-alpha band synchronization across frontal, parietal and visual cortex mediates behavioral and neuronal effects of visuospatial attention . Neuroimage , 165 , 222 – 237 . 10.1016/j.neuroimage.2017.10.044 29074278

[b74] Luck , S. J. ( 1995 ). Multiple mechanisms of visual-spatial attention: Recent evidence from human electrophysiology . Behav Brain Res , 71 , 113 – 123 . 10.1016/0166-4328(95)00041-0 8747179

[b75] Luck , S. J. , Chelazzi , L. , Hillyard , S. A. , & Desimone , R. ( 1997 ). Neural mechanisms of spatial selective attention in areas V1, V2, and V4 of macaque visual cortex . J Neurophysiol , 77 , 24 – 42 . 10.1152/jn.1997.77.1.24 9120566

[b76] Luck , S. J. , Woodman , G. F. , & Vogel , E. K. ( 2000 ). Event-related potential studies of attention . Trends Cogn Sci , 4 , 432 – 440 . 10.1016/S1364-6613(00)01545-X 11058821

[b77] Luo , T. Z. , & Maunsell , J. H. R. ( 2015 ). Neuronal modulations in visual cortex are associated with only one of multiple components of attention . Neuron , 86 , 1182 – 1188 . 10.1016/j.neuron.2015.05.007 26050038 PMC4458699

[b78] Macaluso , E. , & Doricchi , F. ( 2013 ). Attention and predictions: Control of spatial attention beyond the endogenous-exogenous dichotomy . Front Hum Neurosci , 7 , 685 . 10.3389/fnhum.2013.00685 24155707 PMC3800774

[b79] Maeder , C. L. , Sannelli , C. , Haufe , S. , & Blankertz , B. ( 2012 ). Pre-stimulus sensorimotor rhythms influence brain-computer interface classification performance . IEEE Trans Neural Syst Rehabil Eng , 20 , 653 – 662 . 10.1109/TNSRE.2012.2205707 22801528

[b80] Marzecová , A. , Schettino , A. , Widmann , A. , SanMiguel , I. , Kotz , S. A. , & Schröger , E. ( 2018 ). Attentional gain is modulated by probabilistic feature expectations in a spatial cueing task: ERP evidence . Sci Rep , 8 , 54 . 10.1038/s41598-017-18347-1 29311603 PMC5758810

[b81] Mathewson , K. E. , Lleras , A. , Beck , D. M. , Fabiani , M. , Ro , T. , & Gratton , G. ( 2011 ). Pulsed out of awareness: EEG Alpha oscillations represent a pulsed inhibition of ongoing cortical processing . Front Psychol , 2 , 99 . 10.3389/fpsyg.2011.00099 21779257 PMC3132674

[b82] Maunsell , J. H. R. ( 2015 ). Neuronal mechanisms of visual attention . Annu Rev Vis Sci , 1 , 373 – 391 . 10.1146/annurev-vision-082114-035431 28532368 PMC8279254

[b83] Mayo , J. P. , & Maunsell , J. H. R. ( 2016 ). Graded neuronal modulations related to visual spatial attention . J Neurosci , 36 , 5353 – 5361 . 10.1523/JNEUROSCI.0192-16.2016 27170131 PMC4863062

[b84] McFarland , D. J. , Miner , L. A. , Vaughan , T. M. , & Wolpaw , J. R. ( 2000 ). Mu and beta rhythm topographies during motor imagery and actual movements . Brain Topogr , 12 , 177 – 186 . 10.1023/a:1023437823106 10791681

[b85] McGinley , M. J. , Vinck , M. , Reimer , J. , Batista-Brito , R. , Zagha , E. , Cadwell , C. R. , Tolias , A. S. , Cardin , J. A. , & McCormick , D. A. ( 2015 ). Waking state: Rapid variations modulate neural and behavioral responses . Neuron , 87 , 1143 – 1161 . 10.1016/j.neuron.2015.09.012 26402600 PMC4718218

[b86] Moratti , S. , Gundlach , C. , Echegaray de , J., & Müller , M. M. ( 2023 ). Distinct patterns of spatial attentional modulation of steady-state visual evoked magnetic fields (SSVEFs) in subdivisions of the human early visual cortex . Psychophysiology , 61 ( 2 ), e14452 . 10.1111/psyp.14452 37787386

[b87] Morrow , A. , Elias , M. , & Samaha , J. ( 2023 ). Evaluating the evidence for the functional inhibition account of alpha-band oscillations during preparatory attention . J Cogn Neurosci , 35 , 1195 – 1211 . 10.1162/jocn_a_02009 37255429

[b88] Müller , M. M. , Gundlach , C. , Forschack , N. , & Brummerloh , B. ( 2018 ). It takes two to tango: Suppression of task-irrelevant features requires (spatial) competition . Neuroimage , 178 , 485 – 492 . 10.1016/j.neuroimage.2018.05.073 29860080

[b89] Müller , M. M. , & Hillyard , S. ( 2000 ). Concurrent recording of steady-state and transient event-related potentials as indices of visual-spatial selective attention . Clin Neurophysiol , 111 , 1544 – 1552 . 10.1016/S1388-2457(00)00371-0 10964063

[b90] Müller , M. M. , Picton , T. W. , Valdes-Sosa , P. , Riera , J. , Teder-Sälejärvi , W. A. , & Hillyard , S. A. ( 1998 ). Effects of spatial selective attention on the steady-state visual evoked potential in the 20–28 Hz range . Cogn Brain Res , 6 , 249 – 261 . 10.1016/S0926-6410(97)00036-0 9593922

[b91] Müller , M. M. , Teder , W. , & Hillyard , S. A. ( 1997 ). Magnetoencephalographic recording of steady-state visual evoked cortical activity . Brain Topogr , 9 , 163 – 168 . 10.1007/BF01190385 9104827

[b92] Müller , M. M. , Teder-Sälejärvi , W. , & Hillyard , S. A. ( 1998 ). The time course of cortical facilitation during cued shifts of spatial attention . Nat Neurosci , 1 , 631 – 634 . 10.1038/2865 10196572

[b93] Norcia , A. M. , Appelbaum , L. G. , Ales , J. M. , Cottereau , B. R. , & Rossion , B. ( 2015 ). The steady-state visual evoked potential in vision research: A review . J Vis , 15 , 4 . 10.1167/15.6.4 PMC4581566 26024451

[b94] Nuttall , R. , Jäger , C. , Zimmermann , J. , Archila-Melendez , M. E. , Preibisch , C. , Taylor , P. , Sauseng , P. , Wohlschläger , A. , Sorg , C. , & Dowsett , J. ( 2022 ). Evoked responses to rhythmic visual stimulation vary across sources of intrinsic alpha activity in humans . Sci Rep , 12 , 5986 . 10.1038/s41598-022-09922-2 35396521 PMC8993822

[b95] Ogata , K. , Nakazono , H. , Uehara , T. , & Tobimatsu , S. ( 2019 ). Prestimulus cortical EEG oscillations can predict the excitability of the primary motor cortex . Brain Stimul , 12 , 1508 – 1516 . 10.1016/j.brs.2019.06.013 31235367

[b96] Orf , M. , Wöstmann , M. , Hannemann , R. , & Obleser , J. ( 2023 ). Target enhancement but not distractor suppression in auditory neural tracking during continuous speech . iScience , 25 ( 6 ), 106849 . 10.1016/j.isci.2023.106849 PMC10251127 37305701

[b97] Pastor , M. A. , Valencia , M. , Artieda , J. , Alegre , M. , & Masdeu , J. C. ( 2007 ). Topography of cortical activation differs for fundamental and harmonic frequencies of the steady-state visual-evoked responses. An EEG and PET H215O study . Cereb Cortex , 17 , 1899 – 1905 . 10.1093/cercor/bhl098 17060366

[b98] Peylo , C. , Hilla , Y. , & Sauseng , P. ( 2021 ). Cause or consequence? Alpha oscillations in visuospatial attention . Trends Neurosci , 44 , 705 – 713 . 10.1016/j.tins.2021.05.004 34167840

[b99] Pfurtscheller , G. , & Neuper , C. ( 1997 ). Motor imagery activates primary sensorimotor area in humans . Neurosci Lett , 239 , 65 – 68 . 10.1016/s0304-3940(97)00889-6 9469657

[b100] Pilipenko , A. , & Samaha , J. ( 2024 ). Double dissociation of spontaneous alpha-band activity and pupil-linked arousal on additive and multiplicative perceptual gain . J Neurosci , 44 , e1944232024 . 10.1523/JNEUROSCI.1944-23.2024 38548339 PMC11079969

[b101] Posner , M. I. ( 1980 ). Orienting of attention . Q J Exp Psychol , 32 , 3 – 25 . 10.1080/00335558008248231 7367577

[b102] R Core Team . ( 2016 ). R: A Language and Environment for Statistical Computing . R Foundation for Statistical Computing . https://www.r-project.org/

[b103] Regan , D. ( 1989 ). Human brain electrophysiology: Evoked potentials and evoked magnetic fields in science and medicine . Elsevier .

[b104] Rosenberg , M. D. , Finn , E. S. , Constable , R. T. , & Chun , M. M. ( 2015 ). Predicting moment-to-moment attentional state . Neuroimage , 114 , 249 – 256 . 10.1016/j.neuroimage.2015.03.032 25800207

[b105] Rowland , J. M. , Plas van der , L. T. , Loidolt , M. , Lees , R. M. , Keeling , J. , Dehning , J. , Akam , T. , Priesemann , V. , & Packer , A. M. ( 2023 ). Propagation of activity through the cortical hierarchy and perception are determined by neural variability . Nat Neurosci , 26 , 1584 – 1594 . 10.1038/s41593-023-01413-5 37640911 PMC10471496

[b106] Samaha , J. , Iemi , L. , Haegens , S. , & Busch , N. A. ( 2020 ). Spontaneous brain oscillations and perceptual decision-making . Trends Cogn Sci , 24 ( 8 ), 639 – 653 . 10.1016/j.tics.2020.05.004 32513573

[b107] Samaha , J. , Iemi , L. , & Postle , B. R. ( 2017 ). Prestimulus alpha-band power biases visual discrimination confidence, but not accuracy . Conscious Cogn , 54 , 47 – 55 . 10.1016/j.concog.2017.02.005 28222937 PMC5561529

[b108] Samaha , J. , Sprague , T. C. , & Postle , B. R. ( 2016 ). Decoding and reconstructing the focus of spatial attention from the topography of alpha-band oscillations . J Cogn Neurosci , 28 , 1090 – 1097 . 10.1162/jocn_a_00955 27003790 PMC5074376

[b109] Sauseng , P. , Klimesch , W. , Stadler , W. , Schabus , M. , Doppelmayr , M. , Hanslmayr , S. , Gruber , W. R. , & Birbaumer , N. ( 2005 ). A shift of visual spatial attention is selectively associated with human EEG alpha activity . Eur J Neurosci , 22 , 2917 – 2926 . 10.1111/j.1460-9568.2005.04482.x 16324126

[b110] Schettino , A. , Porcu , E. , Gundlach , C. , Keitel , C. , & Müller , M. M. ( 2020 ). Rapid processing of neutral and angry expressions within ongoing facial stimulus streams: Is it all about isolated facial features? PLoS One , 15 , e0231982 . 10.1371/journal.pone.0231982 32330160 PMC7182236

[b111] Schölvinck , M. L. , Saleem , A. B. , Benucci , A. , Harris , K. D. , & Carandini , M. ( 2015 ). Cortical state determines global variability and correlations in visual cortex . J Neurosci , 35 , 170 – 178 . 10.1523/JNEUROSCI.4994-13.2015 25568112 PMC4287140

[b112] Siegel , M. , Donner , T. H. , Oostenveld , R. , Fries , P. , & Engel , A. K. ( 2008 ). Neuronal synchronization along the dorsal visual pathway reflects the focus of spatial attention . Neuron , 60 , 709 – 719 . 10.1016/j.neuron.2008.09.010 19038226

[b113] Singmann , H. , Bolker , B. , Westfall , J. , Aust , F. , & Ben-Shachar , M. S. ( 2020 ). Afex: Analysis of factorial experiments v. 0.28-1. 10.32614/CRAN.package.afex

[b114] Slagter , H. A. , Prinssen , S. , Reteig , L. C. , & Mazaheri , A. ( 2016 ). Facilitation and inhibition in attention: Functional dissociation of pre-stimulus alpha activity, P1, and N1 components . Neuroimage , 125 , 25 – 35 . 10.1016/j.neuroimage.2015.09.058 26436713

[b115] Sokoliuk , R. , Mayhew , S. D. , Aquino , K. M. , Wilson , R. , Brookes , M. J. , Francis , S. T. , Hanslmayr , S. , & Mullinger , K. J. ( 2019 ). Two spatially distinct posterior alpha sources fulfill different functional roles in attention . J Neurosci , 39 , 7183 – 7194 . 10.1523/JNEUROSCI.1993-18.2019 31341028 PMC6733553

[b116] Stolk , A. , Brinkman , L. , Vansteensel , M. J. , Aarnoutse , E. , Leijten , F. S. S. , Dijkerman , C. H. , Knight , R. T. , Lange de , P. F. , & Toni , I. ( 2019 ). Electrocorticographic dissociation of alpha and beta rhythmic activity in the human sensorimotor system . Elife , 8 , e48065 . 10.7554/eLife.48065 31596233 PMC6785220

[b117] Störmer , V. S. , Alvarez , G. A. , & Cavanagh , P. ( 2014 ). Within-hemifield competition in early visual areas limits the ability to track multiple objects with attention . J Neurosci , 34 , 11526 – 11533 . 10.1523/JNEUROSCI.0980-14.2014 25164651 PMC4145167

[b118] Strauß , A. , Wöstmann , M. , & Obleser , J. ( 2014 ). Cortical alpha oscillations as a tool for auditory selective inhibition . Front Hum Neurosci , 8 , 350 . 10.3389/fnhum.2014.00350 24904385 PMC4035601

[b119] Thies , M. , Zrenner , C. , Ziemann , U. , & Bergmann , T. O. ( 2018 ). Sensorimotor mu-alpha power is positively related to corticospinal excitability . Brain Stimul , 11 , 1119 – 1122 . 10.1016/j.brs.2018.06.006 29945791

[b120] Thut , G. , Nietzel , A. , Brandt , S. A. , & Pascual-Leone , A. ( 2006 ). Alpha-band electroencephalographic activity over occipital cortex indexes visuospatial attention bias and predicts visual target detection . J Neurosci , 26 , 9494 – 9502 . 10.1523/JNEUROSCI.0875-06.2006 16971533 PMC6674607

[b121] Tune , S. , Alavash , M. , Fiedler , L. , & Obleser , J. ( 2021 ). Neural attentional-filter mechanisms of listening success in middle-aged and older individuals . Nat Commun , 12 , 1 – 14 . 10.1038/s41467-021-24771-9 34312388 PMC8313676

[b122] Tune , S. , Wöstmann , M. , & Obleser , J. ( 2018 ). Probing the limits of alpha power lateralisation as a neural marker of selective attention in middle-aged and older listeners . Eur J Neurosci , 48 , 2537 – 2550 . 10.1111/ejn.13862 29430736

[b123] Van Diepen , R. , Foxe , J. J. , & Mazaheri , A. ( 2019 ). The functional role of alpha-band activity in attentional processing: The current zeitgeist and future outlook . Curr Opin Psychol , 29 , 229 – 238 . 10.1016/j.copsyc.2019.03.015 31100655

[b124] van Ede , F. , de Lange , F. , Jensen , O. , & Maris , E. ( 2011 ). Orienting attention to an upcoming tactile event involves a spatially and temporally specific modulation of sensorimotor alpha- and beta-band oscillations . J Neurosci , 31 , 2016 – 2024 . 10.1523/JNEUROSCI.5630-10.2011 21307240 PMC6633042

[b125] VanRullen , R. ( 2018 ). Attention cycles . Neuron , 99 ( 4 ), 632 – 634 . 10.1016/j.neuron.2018.08.006 30138586

[b126] Vinck , M. , Batista-Brito , R. , Knoblich , U. , & Cardin , J. A. ( 2015 ). Arousal and locomotion make distinct contributions to cortical activity patterns and visual encoding . Neuron , 86 , 740 – 754 . 10.1016/j.neuron.2015.03.028 25892300 PMC4425590

[b127] Voytek , B. , Samaha , J. , Rolle , C. E. , Greenberg , Z. , Gill , N. , Porat , S. , Kader , T. , Rahman , S. , Malzyner , R. , & Gazzaley , A. ( 2017 ). Preparatory encoding of the fine scale of human spatial attention . J Cogn Neurosci , 29 , 1302 – 1310 . 10.1162/jocn_a_01124 28294717 PMC7474864

[b128] Wagenmakers , E.-J. , & Brown , S. ( 2007 ). On the linear relation between the mean and the standard deviation of a response time distribution . Psychol Rev , 114 , 830 – 841 . 10.1037/0033-295X.114.3.830 17638508

[b129] Walter , S. , Quigley , C. , & Mueller , M. M. ( 2014 ). Competitive interactions of attentional resources in early visual cortex during sustained visuospatial attention within or between visual hemifields: Evidence for the different-hemifield advantage . J Cogn Neurosci , 26 , 938 – 954 . 10.1162/jocn_a_00547 24345166

[b130] Waschke , L. , Kloosterman , N. A. , Obleser , J. , & Garrett , D. D. ( 2021 ). Behavior needs neural variability . Neuron , 109 , 751 – 766 . 10.1016/j.neuron.2021.01.023 33596406

[b131] Wiesman , A. I. , & Wilson , T. W. ( 2020 ). Attention modulates the gating of primary somatosensory oscillations . Neuroimage , 211 , 116610 . 10.1016/j.neuroimage.2020.116610 32044438 PMC7111587

[b132] Worden , M. S. , Foxe , J. J. , Wang , N. , & Simpson , G. V. ( 2000 ). Anticipatory biasing of visuospatial attention indexed by retinotopically specific alpha-band electroencephalography increases over occipital cortex . J Neurosci , 20 , RC63 . 10.1523/JNEUROSCI.20-06-j0002.2000 10704517 PMC6772495

[b133] Wöstmann , M. , Alavash , M. , & Obleser , J. ( 2019 ). Alpha oscillations in the human brain implement distractor suppression independent of target selection . J Neurosci , 39 , 9797 – 9805 . 10.1523/JNEUROSCI.1954-19.2019 31641052 PMC6891068

[b134] Wöstmann , M. , Herrmann , B. , Maess , B. , & Obleser , J. ( 2016 ). Spatiotemporal dynamics of auditory attention synchronize with speech . Proc Natl Acad Sci USA , 113 , 3873 – 3878 . 10.1073/pnas.1523357113 27001861 PMC4833226

[b135] Wyart , V. , Myers , N. E. , & Summerfield , C. ( 2015 ). Neural mechanisms of human perceptual choice under focused and divided attention . J Neurosci , 35 , 3485 – 3498 . 10.1523/JNEUROSCI.3276-14.2015 25716848 PMC4402727

[b136] Yamashita , A. , Rothlein , D. , Kucyi , A. , Valera , E. M. , & Esterman , M. ( 2021 ). Brain state-based detection of attentional fluctuations and their modulation . Neuroimage , 236 , 118072 . 10.1016/j.neuroimage.2021.118072 33882346

[b137] Zemon , V. , & Ratliff , F. ( 1982 ). Visual evoked potentials: Evidence for lateral interactions . Proc Natl Acad Sci USA , 79 , 5723 – 5726 . 10.1073/pnas.79.18.5723 6957888 PMC346977

[b138] Zemon , V. , & Ratliff , F. ( 1984 ). Intermodulation components of the visual evoked potential: Responses to lateral and superimposed stimuli . Biol Cybern , 50 , 401 – 408 . 10.1007/bf00335197 6487677

[b139] Zhigalov , A. , & Jensen , O. ( 2020 ). Alpha oscillations do not implement gain control in early visual cortex but rather gating in parieto-occipital regions . Hum Brain Mapp , 41 , 5176 – 5186 . 10.1002/hbm.25183 32822098 PMC7670647

[b140] Zrenner , C. , Belardinelli , P. , Ermolova , M. , Gordon , P. C. , Stenroos , M. , Zrenner , B. , & Ziemann , U. ( 2022 ). µ-rhythm phase from somatosensory but not motor cortex correlates with corticospinal excitability in EEG-triggered TMS . J Neurosci Methods , 379 , 109662 . 10.1016/j.jneumeth.2022.109662 35803405

