# Supplementary material for: Alpha-band fluctuations represent behaviorally relevant excitability changes as a consequence of top–down guided spatial attention in a probabilistic spatial cueing design
Source: Imaging Neurosci (Camb). 2024 Oct 15;2:imag-2-00312. doi: 10.1162/imag_a_00312 (PMC12290535; doi:10.1162/imag_a_00312)
Supplement: Supplementary Material [file imag_a_00312-supp.pdf]

Supplemental information to: Alpha-band fluctuations represent behaviorally relevant excitability changes as a consequence of top-down guided spatial attention in a probabilistic spatial cueing design

Analysis of Signal Coherence between Visual and Motor Alpha-band Activity

The analyses in the main manuscript revealed cue-validity-related modulations of visual and motor alpha-band activity as well as different patterns of predictive relationships with single-trial reaction times. In order to test whether or not both alpha-band signals measured at these sites have distinct neural generators and are not mainly based on either volume conduction of the same signal or represent a single dipole projecting to central and visual leads we implemented a signal coherence analysis.

For this purpose, for each participant, we took the preprocessed single-trial CSD-transformed data, as described in the main manuscript. Signals were then averaged across electrodes within electrode clusters of interest used in previous analyses representing left (P5; P7; P9; PO3; PO7; O1; I1) and right (P6; P8; P10; PO4; PO8; O2; I2) visual as well as motor-alpha band activity (C3; CP3 and C4; CP4). Averaged single-trial time courses from -1500 to 3250 ms around the cue were then complex-Gabor transformed (Frequency-Width-Half-Mean (FWHM) bandwidth of filter: $FWHM_{frequency}$ = ±1 Hz; $FWHM_{time}$ = ±220.636 ms; frequency range from 8 to 14 Hz in steps of 0.25 Hz) and averaged across frequencies. For comparison and evaluation of the results, signals from two neighboring parieto-occipital channels (PO7 and O1), which should show strong signal coherence due to their proximity, were also complex Gabor-transformed in a first step. Separately for each experimental condition (valid left cue,

valid right cue, invalid left cue, invalid right cue, neutral cue), phase locking value (PLV) time courses were calculated across trials for each sample/time point according to the formula:

$$PLV_{sample} = \left|\frac{1}{N}\sum_{n=1}^{N} e^{i(x-y)}\right| \qquad (1)$$

n = index of trial number; x, y = instantaneous phase values of the Gabor signals from different electrode clusters; N = total number of trials

These PLV time courses represent a measure of the stability of the phase relationship of alpha-band activity at motor and visual electrode clusters across trials for each of the time points. For each time point, PLV 95% CIs were calculated based on a null distribution of 10,000 pseudo PLVs for which no relationship between the signals of interest were expected. This null distribution was created by randomly drawing x and y values from different trials without replacement and thus calculating the coherence between unrelated signals measured at different time points 10,000 times. Derived first-level 95%-CIs were averaged across participants to yield approximate CIs of PLVs for non-coherently modulated signals. In addition, the instantaneous phase values as well as the phase value differences for all signal pairs were calculated and mean phase angles and dispersions were derived. Crucially, we assumed that if signals had the same neural generator and were measured at different electrodes due to volume conduction, the distribution of phase difference values would be expected to be centered at 0° phase difference. The divergence of phase difference values from uniformity was tested via Herrmans-Rasson test (Hermans & Rasson, 1985; Landler et al., 2018) implemented in matlab (Cloherty, 2023). To test whether potentially non-uniformly distributed phase-differences differ from a direction of 0°, we additionally ran a circular v-test, implemented in the Circular Statistics Toolbox in matlab (Berens, 2009).

As visible in Supp. Figure 1, the analysis revealed different PLV dynamics for the different combinations of regions of interest. While PLVs for alpha-band activity between visual and motor regions was overall lower during valid cueing conditions as compared to all other conditions (see Supp. Figure 1 A), only PLVs measured during valid cueing conditions were clearly distinct from CIs (in green) for PLVs calculated for random data, while for other

conditions measured PLVs fell into the PLV Cis (in red and purple) of random data (see Supp. Figure 1 B). For neighboring parieto-occipital regions PO7 and O1, supposedly capturing alpha-band signals from the same generators, PLVs were in general higher and clearly distinct from CIs of PLVs for random data. In the same vein, the distribution of phase differences for alpha-band signals measured between regions of interest in the pre-cue time window (-1000 to 0 ms) was different for visual and motor alpha-band activity as compared to signals measured at neighboring channels (see Supp. Figure 1 C,D). For PLV measures between left visual and motor alpha band activity the phase difference was on average 177.475° (*SD* = 50.046) across participants. The Herrmans-Rasson test found difference values to be non-uniformly distributed (T = 2200.056, p = .001). In addition, the circular v-test, did not provide any evidence for a non-uniform distribution of phase differences centered at 0° (v = -17.302, p = .999). The same pattern was found for the phase relationship between right visual and motor alpha-band activity. With an average phase difference of 159.657° (*SD* = 61.522), the Herrmans-Rasson test points towards non-uniform distribution (T = 2354.614, p =.001) Again there was no evidence for a non-uniform distribution of phase differences centered at 0°, based on the circular v-test (v = -11.119, p =.999). For alpha-band signals recorded from neighboring parieto-occipital channels, the average difference was 0.987° (*SD* = 25.476), with the Herrmans-Rasson test pointing towards a non-uniform distribution (T = 1110.361, p < .001). The circular v-test suggest that the alternative hypothesis of a non-uniform distribution centered at 0° should be accepted (v = 25.228, p < .001). The overall pattern points towards some phase coherence between visual and motor alpha-band activity with, however, phase differences deviating from zero and, thus, suggesting that alpha-band activity recorded from motor and parieto-occipital channels indeed have different neural generators.

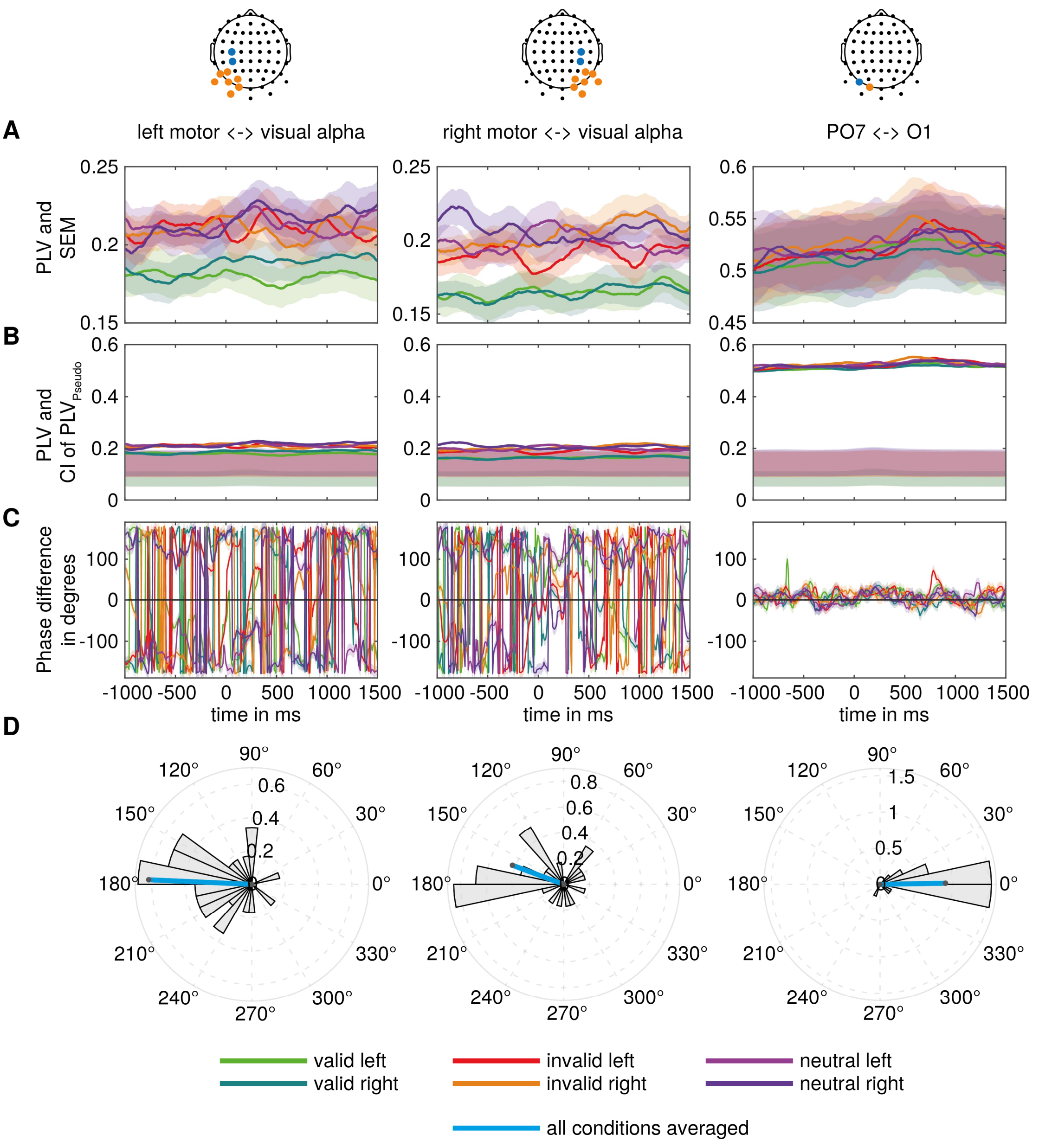

Supp. Figure 1 ***Results of PLV Analysis*** Main findings of phase locking analysis for alpha band-activity recorded between different electrodes and clusters as depicted on top. **A)** Time resolved Phase-locking values (PLV) are depicted averaged across participants separately for different experimental conditions. Shaded area represents SEM. **B)** PLV values are again depicted with shaded area now representing 95% CI of Pseudo-PLV values generated by randomly drawing from different trials to depict PLV distributions expected for random signal coherence. **C)** Difference of phase angles between signals, averaged across participants. Shaded area represents SEM. **D)** Probability-Density-Normalized Phase-difference histogram of single subject phase angle differences averaged across conditions and the pre-cue time window (-1000 to 0 ms). The average vector with average direction and resultant vector length is depicted by the blue line.

Relationship of Reaction Times, Cue Validity and Post-target Motor Alpha-band activity

As a sanity check, we tested whether RTs were predicted by single-trial post-target motor-alpha-band amplitudes, which were previously found to be a strong predictor of grip force, gait control and response-time fluctuations (Chen et al., 2013; Haddix et al., 2021; Johari

& Behroozmand, 2020; Zhao et al., 2022). We looked into the relationship between target-response-related motor alpha-band activity recorded in a time window after target presentation (0 to 1000 ms) and single-trial reaction times for correct responses separate for the different cue-validity conditions. The model again captured the general cue-validity effect: the median predicted reaction time being 9.474 ms faster after a valid as compared to a neutral cue (95% HPD interval from 1.044 to 18.405 ms), 30.573 ms faster after a valid as compared an invalid cue (95% HPD interval from 20.882 to 39.765 ms) and 20.860 ms faster for neutrally as compared to invalidly cued targets (95% HPD interval from 12.536 to 29.750 ms).

In addition, for all cue validity levels there was a positive relationship between motor alpha-band activity and reaction times. The lower the post-target alpha-band amplitudes measured above motor cortex of the response hand, i.e., contralateral to the target, the faster the correct button press (see Figure 9). For all cueing conditions, the slope of the relationship was consistently positive (valid: median slope = 5.045; 95% HPD interval from 3.200 to 6.773; neutral: median slope = 5.213; 95% HPD interval from 2.386 to 8.083; invalid: median slope = 3.774; 95% HPD interval from 1.037 to 6.575).

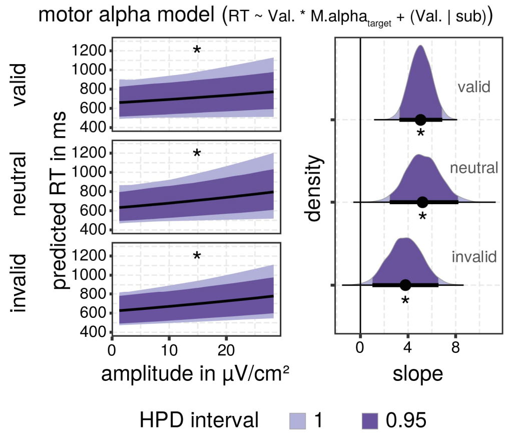

Supp. Figure 2 ***Posterior Distributions of Effects of Post-Target Motor Alpha-band Amplitudes on Reaction Times*** Separately for the cue validity conditions the predicted relationship between post-target alpha-band amplitudes measured above the motor cortex of the response hand (i.e., contralateral to the target event) and reaction times predicted across model draws is displayed. The 95 % highest posterior density (HPD) interval for the predicted relationship across model draws is depicted by the ribbon of the saturated color. The median of the distribution is indexed by the black line. The respective density distribution of predicted slope values is plotted on the right side with the 95 % HDP interval again depicted by the saturated color and the bold horizontal line at the bottom of each distribution. Median slopes are indexed by a black circle. * marks the distributions for which all predicted slopes within the 95 % HDP interval are different from zero and the marginal effect is consistent across model draws.

Relationship of Post-cue Electrophysiological Measures and Attention

In addition to the conventional analysis, we evaluated whether and how much the electrophysiological measures themselves were modulated by attention in the post-cue time window on the basis of the Bayesian multilevel modeling approach. As in the analysis of reaction times, maximally complex models were constructed with post-cue (500 to 1500 ms) amplitudes of the respective electrophysiological measures (SSVEPs: `SSVEP ~ Cue * V.Alpha + (Cue | sub)`, visual alpha: `V.Alpha ~ Cue + (Cue | sub)`, and motor alpha-band activity: `M.Alpha ~ Cue + (Cue | sub)`) now representing the to-be-predicted response and the attentional cue (cued, neutral, uncued) representing the potential predicting factor. For the SSVEP model, we additionally included visual alpha-band amplitudes recorded contralateral to the target as an additional predictor to re-evaluate whether both neural measures were related (see Antonov et al., 2020; Gundlach et al., 2020; Zhigalov & Jensen, 2020). The model evaluations of the marginal effects were based on the same logic as described for the reaction time models to test whether single-trial neurophysiological measures were substantially modulated by the attentional cue.

As visible in Supp Figure 3, all neurophysiological signals (SSVEP, visual alpha-band and motor alpha-band activity contralateral to the target) measured 500 to 1500 ms post-cue were modulated by attention, i.e., the factor cue (cued, uncued, neutral). Contrasts between Cue levels revealed different patterns for the different neural measures. Predicted posterior SSVEPs amplitudes for stimuli were higher when they were cued than when they were uncued (median difference: 0.152 µV/cm² 95% HPD interval from 0.030 to 0.273 µV/cm²). Neutrally cued stimuli were also higher in amplitude than uncued stimuli (median difference: 0.181 µV/cm² 95% HPD interval from 0.062 to 0.294 µV/cm²). There was no unequivocal direction of differences between amplitudes for cued and neutrally cued stimuli across all model draws and they were thus comparable (median difference: -0.029 µV/cm² 95% HPD interval from -0.149 to 0.076 µV/cm²). Visual alpha-band amplitudes were lower contralateral to cued as compared to uncued stimuli (median difference: -0.204 µV/cm² 95% HPD interval from -0.320 to -0.090 µV/cm²), while amplitudes contralateral to neutrally cued stimuli did not unambiguously differ

from cued (median difference: -0.053 µV/cm² 95% HPD interval from -0.150 to 0.040 µV/cm²) and uncued stimuli (median difference: -0.149 µV/cm² 95% HPD interval from -0.311 to 0.012 µV/cm²). Motor alpha-band amplitude was higher contralateral to the uncued as compared to the cued stimulus (median difference: 0.152 µV/cm² 95% HPD interval from 0.030 to 0.273 µV/cm²) and as compared to a neutrally cued stimulus (median difference: 0.181 µV/cm² 95% HPD interval from 0.062 to 0.294 µV/cm²). Motor alpha amplitudes did not unambiguously differ contralateral to neutrally cued and cued stimuli across model draws (median difference: -0.029 µV/cm² 95% HPD interval from -0.149 to 0.076 µV/cm²). For the SSVEP model family, we also included alpha-band amplitudes as a potential predictor. This allowed us to (re-) test a previous finding that SSVEP amplitudes and alpha-band activity do not covary on a trial-by-trial basis in spatial attention tasks (Antonov et al., 2020; Gundlach et al., 2020; Zhigalov & Jensen, 2020). The analysis of the posterior predicted slopes again revealed no consistent relationship between visual alpha-band amplitudes and SSVEPs for the different cueing conditions (cued: median slope = 0.014, 95 % HDP interval from -0.010 to 0.036; neutral: median slope = 0.027, 95 % HDP interval from -0.001 to 0.054; uncued: median slope = 0.017, 95 % HDP interval from -0.002 to 0.037), suggesting an independent modulation of single trial alpha-band amplitudes and SSVEP amplitudes by spatial attention.

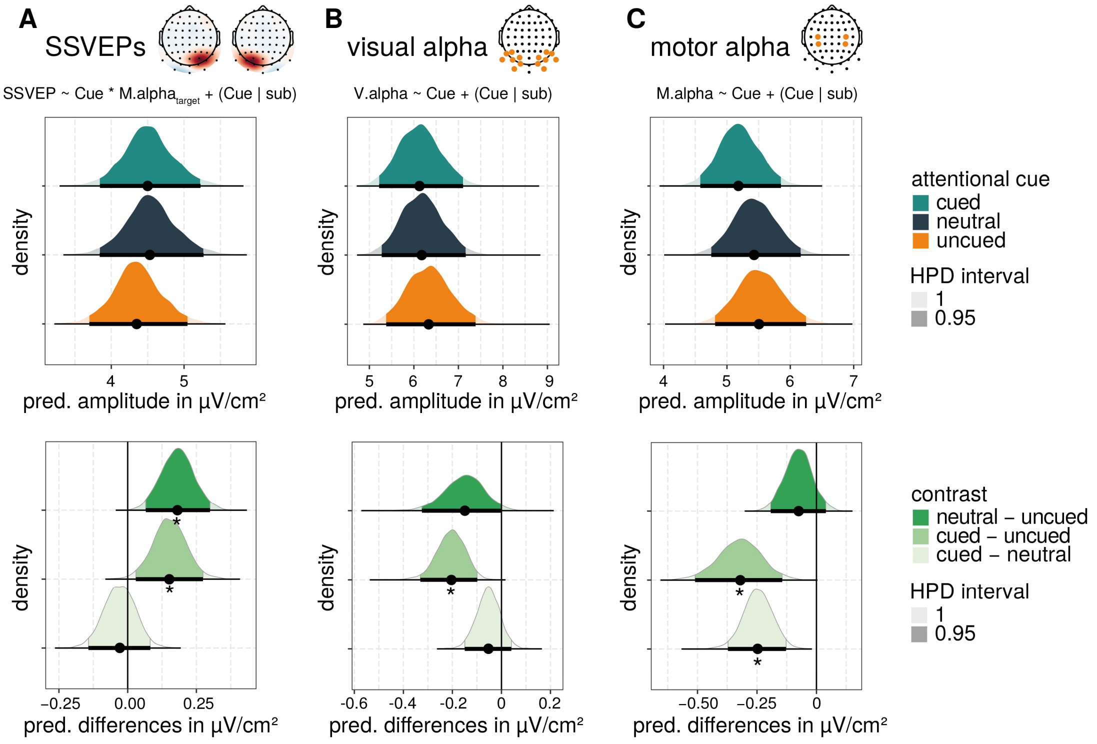

Supp. Figure 3 ***Illustration of Marginal Effects of Attentional Cue on Amplitudes of Neural Measures*** A) Top: Display of posterior distribution of median SSVEP amplitudes predicted by each model draw separately for different attentional cues. 95% posterior density (HPD) intervals are indicated by bright colors and the bold horizontal lines. Median amplitudes are indexed by black circles. Similarly, posterior density plots for paired contrasts between predicted values of different attentional cue levels are displayed at the bottom. * marks the distributions for which all predicted contrasts within the 95 % HDP interval are different from zero and the marginal effect is consistent across model draws. B) Predicted Median visual alpha-band and C) motor alpha-band amplitudes are displayed with the same convention as in A).

Relationship of Post-Cue z-scored Electrophysiological Measures and Reaction Times

In our main analysis presented in the manuscript, we examined whether the cue validity and single-trial neurophysiological measures were predictive of single-trial reaction times by including them as potential predictors in Bayesian multilevel regression models. Both predictors were, however, not independent as we also showed that the cue affected our neurophysiological measures. Multicollinearity of predictors in regression models, i.e., a strong relationship between both, may pose a threat to the interpretation and fit of the models (McElreath, 2016). This issue may arise in the case of a strong association between predictors, which, given the respective effect sizes depicted for the repeated measures ANOVAe and the Bayesian R^2 factors extracted from the brms models, this may not be the case for our models. We, however, ran a control analysis to evaluate the results in relationship to findings for which

multicollinearity was ruled out. For this purpose, we fitted another set of Bayesian Multilevel Models the same way as described in the manuscript with the addition that the post-cue single-trial neurophysiological measures were initially z-standardized for each participant and cueing condition (valid, neutral and invalid). By doing so, average single-trial amplitude measures did not differ between cueing conditions. This allowed us to evaluate whether in addition to the cue validity, post-cue single trial amplitudes of the different neural measures represent a potential predictor for reaction times. The analysis of the distributions of the marginal posterior effects mirrored the results presented in the manuscript. Model fits converged with rhat values generally being 1.01 or lower. As visible in Supp. Figure 4 and Supp. Table 1, z-standardized SSVEP amplitudes did not predict reaction times consistently across model draws, as the 95% HPD interval of the distribution of the predicted slopes spanned from negative to positive values. Similarly, z-standardized post-cue motor alpha-band amplitudes were no consistent predictor. As for the analysis in the main manuscript, z-standardized visual alpha-band amplitudes were predictive of reaction times. In valid and neutral trials, lower alpha-band amplitudes contralateral to the ring with the upcoming stimulus were associated with faster reaction times and additionally, in valid trials, higher alpha-band amplitudes contralateral to the ring without the **target** (or ipsilateral to the ring with the upcoming target) were predictive of faster reaction times. Overall, the same set of predictors was found in the control analysis for z-standardized neural measures, suggesting that multicollinearity between the potential predictors does not pose a problem for the analysis of our data and the interpretation of the predictors.

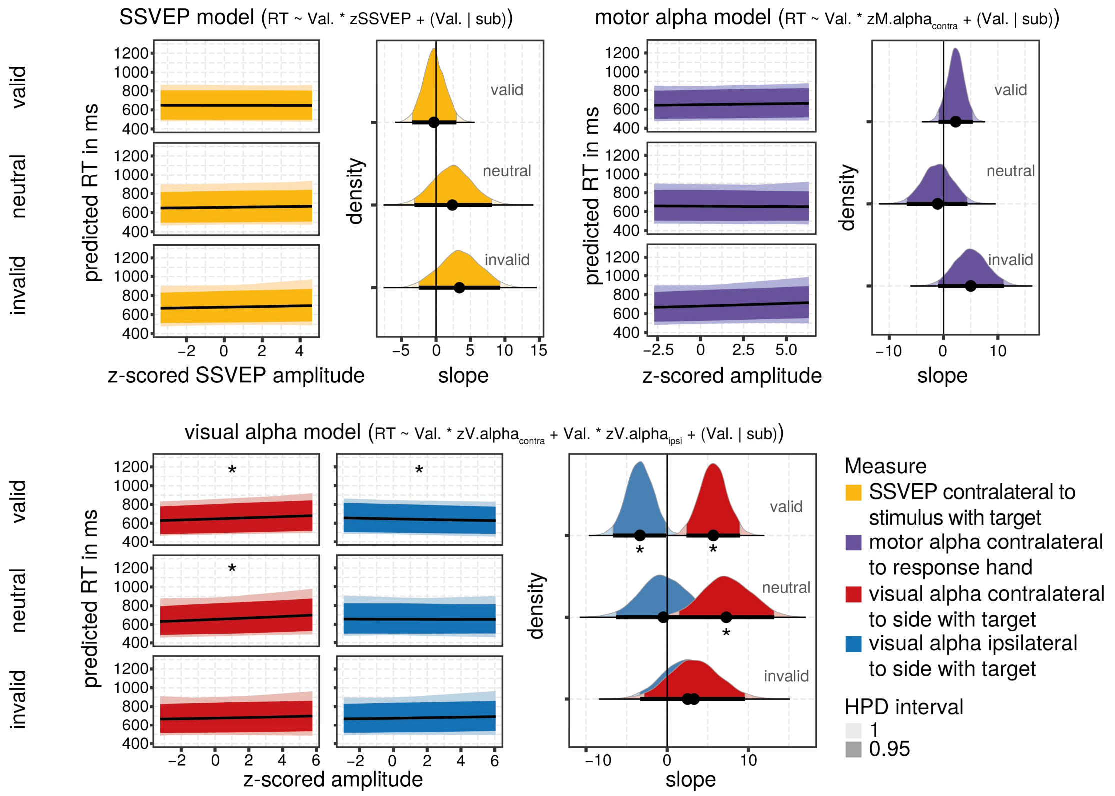

Supp. Figure 4 Posterior Distributions of Effects of z-scored Post-Cue Electrophysiological Measures on Reaction Times The predicted relationship between the amplitude of z-scored post-cue electrophysiological measures and reaction times is displayed separately for each cue validity condition and measure. The 95 % highest posterior density (HPD) interval for the predicted relationship across model draws is depicted by the ribbon of the saturated color. The median of the distribution is indexed by the black line. The respective density distribution of predicted slope values is plotted on the right side with the 95 % HDP intervals depicted by the saturated colors and bold horizontal lines at the bottom of each distribution. Median slopes are indexed by a black circle. * marks the distributions for which all predicted slopes within the 95 % HDP interval are different from zero.

Supp. Table 1 Marginal posterior summaries for predicted slopes of the relationship between the z-scored amplitude of post-cue neural measures and RTs for Cue-Validity conditions, respectively

Neural Measure	Cue Validity	Predicted Slope		
		Median	2.5 % HPD	97.5 % HPD
**SSVEP**	valid	-0.322	-3.238	3.126
	neutral	2.360	-3.126	8.118
	invalid	3.416	-2.113	9.614
Visual alpha	***valid***	***5.679***	***2.414***	***8.992***
contralateral	***neutral***	***7.264***	***1.265***	***12.906***
	invalid	3.319	-2.979	9.363
Visual alpha	***valid***	***-3.349***	***-6.500***	***-0.070***
ipsilateral	neutral	-0.499	-6.554	5.137
	invalid	2.522	-3.486	8.510
**Motor alpha**	valid	2.230	-0.787	5.463
**contralateral**	neutral	-1.085	-7.038	4.100
	invalid	4.998	-0.717	11.331

Note: Substantial marginal effects, i.e. for which all slopes in the 95 % Highest Posterior Density (HPD) interval are different from zero, are in bold italics.

Relationship of Visual Alpha-band Amplitudes, SSVEPs and Reaction Times estimated in a combined model

We were interested in the combined contribution of visual alpha-band activity and SSVEPs for predicting reaction times. Even though SSVEPs were found being unpredictive for reaction times, we exploratively tested in a combined model, whether or not both neural measures in combination are found to be predictive. For this purpose a model was fitted with SSVEP amplitudes contralateral to the stimulus with the upcoming target and visual-alpha band amplitudes contra and ipsilateral to the side with the target entered as potential predictors in addition to the cue validity:

$$RT \sim Val. * V.alpha_{contra} + Val. * V.alpha_{ipsi} + Val. * SSVEP + (Val. \mid sub)$$

After checking for model convergence (all Rhats $\leq$ 1.01), as in the analysis described in the manuscript, the distributions of the modeled slopes (and their 95% HPD intervals) of all predictors were extracted separately for all cue-validity levels to evaluate whether factors consistently and substantially predicted single-trial reaction times. Overall the effects of the combined model mirrored the effects of the separate single predictor models (see Supp. Figure 5 and Supp. Table 2).

Supp. Table 2 Marginal posterior summaries for predicted slopes of the relationship between the amplitude of post-cue neural measures and RTs for Cue-Validity conditions, respectively as modelled in a combined model

Neural Measure	Cue Validity	Predicted Slope		
		Median	2.5 % HPD	97.5 % HPD
**SSVEP**	valid	0.028	-1.126	1.088
	neutral	0.678	-1.121	2.503
	invalid	1.865	-0.137	4.116
Visual alpha	***valid***	***2.986***	***1.444***	***4.521***
contralateral	***neutral***	***3.678***	***0.834***	***6.099***
	invalid	0.903	-1.468	3.437
Visual alpha	***valid***	***-1.759***	***-3.058***	***-0.413***
ipsilateral	neutral	-1.005	-3.556	1.549
	invalid	-0.205	-2.839	2.41

Note: Substantial marginal effects, i.e. for which all slopes in the 95 % Highest Posterior Density (HPD) interval are different from zero, are in bold italics.

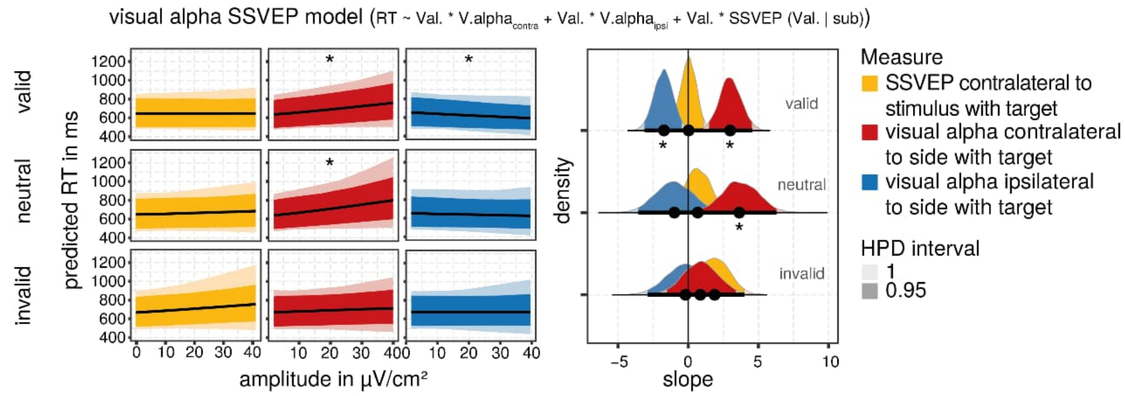

Supp. Figure 5 ***Posterior Distributions of Effects of Visual Alpha-band and SSVEP amplitudes on Reaction Times based on a combined model*** The predicted relationship between the amplitude of z-scored post-cue electrophysiological measures and reaction times is displayed separately for each cue validity condition and measure. The 95 % highest posterior density (HPD) interval for the predicted relationship across model draws is depicted by the ribbon of the saturated color. The median of the distribution is indexed by the black line. The respective density distribution of predicted slope values is plotted on the right side with the 95 % HDP intervals depicted by the saturated colors and bold horizontal lines at the bottom of each distribution. Median slopes are indexed by a black circle. * marks the distributions for which all predicted slopes within the 95 % HDP interval are different from zero.

While in this model visual alpha-band amplitudes and SSVEP amplitudes showed the same predictive pattern as in the respective single models, we still tested whether the combined model is ‘superior’ to the pure alpha-model as it may predict data more accurately. For this purpose, a between-model comparison was based on an approximate leave-one-out cross-validation procedure implemented via the function `loo` as part of the *brms* package (Vehtari et al., 2017). Hereby the prediction accuracy of each model was estimated as the expected log pointwise posteriors distribution (ELPD), based on the existing posterior simulation draws of the model fits and compared between models to find the best-performing models. When comparing the two models ELPD differences as well as their Standard Errors (accounting for the uncertainty of the approximate leave-one-out cross-validation procedure) were calculated. Together with the complexity of the model (i.e., based on the number of factors in each model), ELPD differences formed the basis of the decision for any of the two compared models. This then allowed us to evaluate the contribution of the tested factors alone or in combination for predicting reaction times. As visible in rev_table 2, the predictive accuracy of the combined model was not better than the single model because ELPD differences fell within 2*SE of the distribution.

Supp. Table 3 Results of LOO-based model comparison for different model families with post-cue neural measures

Model	ELPD	Δ_{ELPD}	SE Δ_{ELPD}	R^2
RT ~ Val. * V.alpha$_{target}$ + Val. * V.alpha$_{notarget}$ + (Val. | sub)	-64432.320	0	0	0.192
RT ~ Val. * V.alpha$_{target}$ + Val. * V.alpha$_{notarget}$ + SSVEP + (Val. | sub)	-64432.361	-0.041	1.767	0.192

Note: ELPD = expected log pointwise predictive density; **Δ_{ELPD}** = ELPD difference relative to the most accurate model with the highest ELPD value, displayed in the first row of each model family; the model to be preferred when comparing models of each model family is displayed in bold italics

References

Antonov, P. A., Chakravarthi, R., & Andersen, S. K. (2020). Too little, too late, and in the wrong place: Alpha band activity does not reflect an active mechanism of selective attention. *NeuroImage*, 117006. https://doi.org/10.1016/j.neuroimage.2020.117006

Berens, P. (2009). CircStat: A MATLAB Toolbox for Circular Statistics. *Journal of Statistical Software*, *31*(10). http://www.jstatsoft.org/v31/i10/

Chen, X., Bin, G., Daly, I., & Gao, X. (2013). Event-related desynchronization (ERD) in the alpha band during a hand mental rotation task. *Neuroscience Letters*, *541*, 238–242. https://doi.org/10.1016/j.neulet.2013.02.036

Cloherty, S. (2023). *A Matlab implementation of the Hermans-Rasson test*. https://github.com/cnuahs/hermans-rasson

Gundlach, C., Moratti, S., Forschack, N., & Müller, M. M. (2020). Spatial Attentional Selection Modulates Early Visual Stimulus Processing Independently of Visual Alpha Modulations. *Cerebral Cortex* , *30*(6), 3686–3703. https://doi.org/10.1093/cercor/bhz335

Haddix, C., Al-Bakri, A. F., & Sunderam, S. (2021). Prediction of isometric handgrip force from graded event-related desynchronization of the sensorimotor rhythm. *Journal of Neural Engineering*, *18*(5). https://doi.org/10.1088/1741-2552/ac23c0

Hermans, M., & Rasson, J. P. (1985). A new Sobolev test for uniformity on the circle. *Biometrika*, *72*(3), 698–702. https://doi.org/10.1093/biomet/72.3.698

Johari, K., & Behroozmand, R. (2020). Event-related desynchronization of alpha and beta band neural oscillations predicts speech and limb motor timing deficits in normal aging. *Behavioural Brain Research*, *393*, 112763. https://doi.org/10.1016/j.bbr.2020.112763

Landler, L., Ruxton, G. D., & Malkemper, E. P. (2018). Circular data in biology: advice for effectively implementing statistical procedures. *Behavioral Ecology and Sociobiology*, *72*(8), 128. https://doi.org/10.1007/s00265-018-2538-y

McElreath, R. (2016). *Statistical Rethinking: A Bayesian Course with Examples in R and Stan*. CRC Press. https://market.android.com/details?id=book-mDo0CwAAQBAJ

Vehtari, A., Gelman, A., & Gabry, J. (2017). Practical Bayesian model evaluation using leave-one-out cross-validation and WAIC. *Statistics and Computing*, *27*(5), 1413–1432. https://doi.org/10.1007/s11222-016-9696-4

Zhao, M., Bonassi, G., Samogin, J., Taberna, G. A., Pelosin, E., Nieuwboer, A., Avanzino, L., & Mantini, D. (2022). Frequency-dependent modulation of neural oscillations across the gait cycle. *Human Brain Mapping*, *43*(11), 3404–3415. https://doi.org/10.1002/hbm.25856

Zhigalov, A., & Jensen, O. (2020). Alpha oscillations do not implement gain control in early visual cortex but rather gating in parieto-occipital regions. *Human Brain Mapping*, *41*(18), 5176–5186. https://doi.org/10.1002/hbm.25183
